# The circadian clock orchestrates spermatogonial differentiation and fertilization by regulating retinoic acid signaling in vertebrates

**DOI:** 10.1093/nsr/nwae456

**Published:** 2024-12-11

**Authors:** Taole Liu, Wei He, Zhaomin Zhong, Chenchen Lu, Lianxin Wu, Ziming Wang, William Kojo Smith, Quan Shi, Qiaoming Long, Han Wang

**Affiliations:** Center for Circadian Clocks, Soochow University, Suzhou 215123, China; School of Basic Medical Sciences, Suzhou Medical College, Soochow University, Suzhou 215123, China; Center for Circadian Clocks, Soochow University, Suzhou 215123, China; School of Basic Medical Sciences, Suzhou Medical College, Soochow University, Suzhou 215123, China; Center for Circadian Clocks, Soochow University, Suzhou 215123, China; School of Basic Medical Sciences, Suzhou Medical College, Soochow University, Suzhou 215123, China; Center for Circadian Clocks, Soochow University, Suzhou 215123, China; School of Basic Medical Sciences, Suzhou Medical College, Soochow University, Suzhou 215123, China; Center for Circadian Clocks, Soochow University, Suzhou 215123, China; School of Basic Medical Sciences, Suzhou Medical College, Soochow University, Suzhou 215123, China; Center for Circadian Clocks, Soochow University, Suzhou 215123, China; School of Basic Medical Sciences, Suzhou Medical College, Soochow University, Suzhou 215123, China; Center for Circadian Clocks, Soochow University, Suzhou 215123, China; School of Basic Medical Sciences, Suzhou Medical College, Soochow University, Suzhou 215123, China; Center for Circadian Clocks, Soochow University, Suzhou 215123, China; School of Basic Medical Sciences, Suzhou Medical College, Soochow University, Suzhou 215123, China; Cam-Su Genomic Resource Center, Soochow University, Suzhou 215123, China; Center for Circadian Clocks, Soochow University, Suzhou 215123, China; School of Basic Medical Sciences, Suzhou Medical College, Soochow University, Suzhou 215123, China

**Keywords:** testis, circadian clock, spermatogenesis, fertilization, retinoic acid, zebrafish, mice

## Abstract

The circadian clock generates and maintains ∼24-hour oscillations in almost all organs. The testis, however, remains mysterious, without a clear understanding of its circadian functions. Our time-series transcriptome analysis reveals more than 1000 rhythmically expressed genes in the zebrafish and mouse testes, respectively. Canonical circadian clock genes are rhythmically expressed in Sertoli cells and regulate retinoic acid (RA) production, which is also evidenced by their co-expression with RA synthesis genes in single Sertoli cells. Genetic and pharmacological manipulations and temporal desynchronization revealed that the circadian clock-regulated RA signaling synchronizes spermatogonial differentiation via *zbtb16a* and promotes fertilization via *izumo1* in zebrafish. Our findings indicate that the testicular circadian clock contributes to reproduction in a cell-specific manner through RA signaling, highlighting circadian roles in male fertility.

## INTRODUCTION

Circadian rhythms are generated and maintained by cell-autonomous clocks throughout the body in a tissue-specific or cell-specific manner [1–3]. These intrinsic circadian clocks are driven by interlocked transcription/translation feedback loops in vertebrates and function as the regulatory network in diverse physiology and behavior, allowing for coordinated phases and adaptation to external changes [4–6]. Dysfunctions of the circadian clock globally or tissue-specifically result in arrhythmia and malfunctions in numerous biological processes, including reproduction [1,7]. Circadian disruption has been reported to strongly impair the reproductive capacities of humans and rodents, which is multifactorial and poorly understood [8,9]. In addition to the clock-regulated hypothalamus-pituitary-gonad axis [10], the peripheral clocks in gonads have long been implicated in significantly affecting fertility in both female and male mice [10,11].

Unlike the majority of tissues/organs studied, however, the testis has long been regarded as the exception without circadian regulation, as canonical circadian clock genes display constant or non-rhythmic expression in the testes of vertebrates, including zebrafish (*Danio rerio*) and rodents [12–15]. The mechanisms underpinning this testicular noncyclic property remain unclear, although the continuously differentiating cells [16], DNA condensation [17] and cancer/testis antigen PASD1 [18] have been implicated in contributing to the neutralization of the testicular clock activities, if any.

Here, we set to employ both zebrafish and mice to examine if the testis displays rhythmic activities [2,5,19]. We investigated the regulatory role of the testis clock in spermatogenesis and fertilization in global and Sertoli cell-specific circadian clock mutants and temporally perturbed models in which the clock activities are disrupted, mimicking jetlag or work shift. Our study pinpoints circadian functions in the testis.

## RESULTS

### The zebrafish and mouse testes exhibit a rhythmic transcriptome

**Figure 1.**
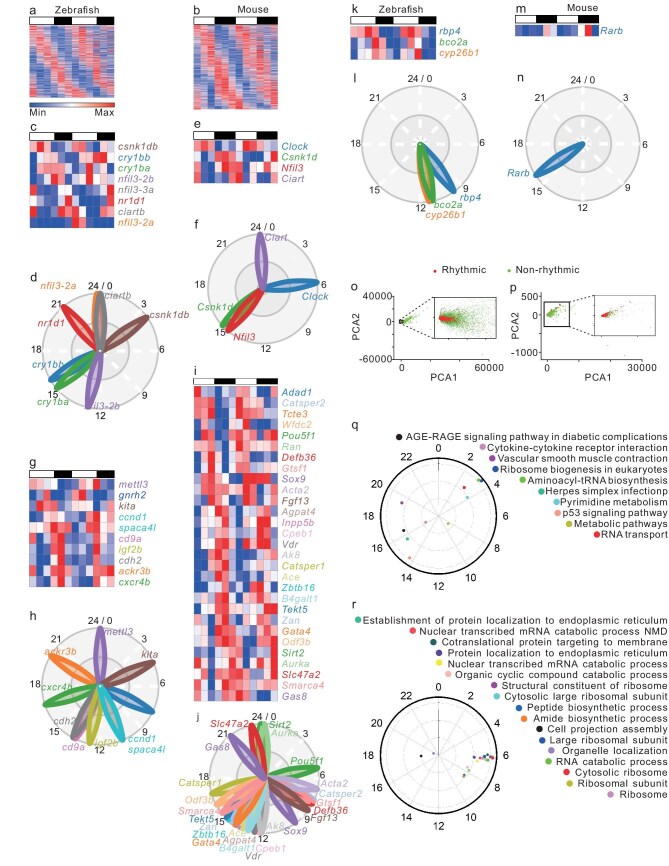
Rhythmic testicular transcriptome in zebrafish and mice. (a and b) Heatmaps of the 1082 zebrafish rhythmic transcripts (a) and 1882 mouse rhythmic transcripts (b), revealed by the MetaCycle analysis of the 12-timepoint transcriptome data (*P* < 0.05, *n* = 2 × 12). (c–n) Heatmaps and phases of the rhythmically expressed zebrafish and mouse genes involved in circadian regulation (zebrafish: c, d; mouse: e and f), testicular functions (zebrafish:g, h; mouse: i, j), and retinoic acid signaling (zebrafish: k, l; mouse: m, n). The gene names (zebrafish: c, g, k; mouse: e, i, m) and their phases (zebrafish: d, h, l; mouse: f, j, n) are color-coded and annotated. (o and p) PCA of all transcripts from the 12 timepoints in the zebrafish (o) and mouse (p) testes shows a single clustering of the rhythmic transcriptome (red dots in the enlarged panel), respectively. (q and r) Phase distribution over the 24-hour cycle of the enriched KEGG pathways in the zebrafish (q) or mouse (r) testes (*P* < 0.05) (Fig. S1).

The circadian clock contributes to life processes and activities by regulating numerous genes encoding key signals and/or functional factors [20,21]. To investigate whether there are rhythmically expressed genes in the vertebrate testis, we conducted transcriptome analysis of the 12-timepoint testes from 2 consecutive days with a 4-hour interval in zebrafish and mice, respectively. Among 23 330 transcripts detected in zebrafish (Table S1) and 23 502 in mice (Table S2), 1082 (4.64%) zebrafish genes (Fig. 1a, and Table S1) and 1882 (8.01%) mouse genes (Fig. 1b, and Table S2), including numerous canonical circadian clock genes (Fig. 1c–f; Tables S1 and S2), as well as other transcriptional factors (Fig. S1a-S1d; Tables S1 and S2), display statistically significant rhythmic expression in the testes, as determined by MetaCycle analysis [22] (*P* < 0.05) (Tables S1 and S2). While *csnk1db, nfil3-2a* and *nfil3-3a* appear most active in the zebrafish testis between ZT0 and ZT4 (Fig. 1d), *Csnk1d* and *Nfil3* peak in the mouse testis around ZT15 (Fig. 1f). In addition, *cry1ba, cry1bb, nfil3-2b* and *nr1d1* peak in the zebrafish testis in the night time (Fig. 1d), while *Clock* peaks in the mouse testis in the early day time (Fig. 1f). Intriguingly, *ciartb* and *Ciart* peak in the zebrafish and mouse testes at ZT0 (Fig. 1d and f), respectively. Notably, numerous genes critical for spermatogenesis and sperm functions are rhythmically expressed in the zebrafish and mouse testes (Fig. 1g–j; Tables S1 and S2). Intriguingly, several genes involved in the retinoic acid (RA) signaling pathway critical for spermatogenesis [23] are also rhythmically expressed in the zebrafish and mouse testes (Fig. 1k–n; Tables S1 and S2). The genes involved in spermatogenesis and sperm functions are transcribed in the zebrafish and mouse testes sequentially during the whole day (Fig. 1g–j), while the genes involved in the RA signaling pathway peak in the evening or early night (Fig. 1k–n), indicative of a rhythmic pattern of endogenous RA in vertebrates. Principal components analysis (PCA) confirmed a cluster of rhythmically expressed genes in the zebrafish and mouse testes (Fig. 1o and p, Tables S1 and S2). Phase set enrichment analysis [24] of various KEGG pathways of these rhythmically expressed genes showed that RNA transport and ribosome biogenesis are highly enriched at noon, metabolic pathways peak in the late afternoon, and other functional pathways are most active in the evening in the zebrafish testis (Fig. 1q); while the top-ranked KEGG pathways, such as the ribosome, ribosomal subunit and mRNA catabolic process, are primarily enriched around ZT6 in the mouse testis (Fig. 1r). Gene ontology (GO) analysis also showed that numerous important cellular functions, including the negative regulation of the RA receptor signaling pathway and sperm flagellum, are enriched in the zebrafish and mouse testes (Fig. S1e-S1f). Together, in sharp contrast to the previously regarded arrhythmic activities of the zebrafish and mouse testes, these results indicate that many aspects of the testicular functions display rhythmicity in vertebrates.

### The testis clock operates in a cell-specific manner in zebrafish

**Figure 2.**
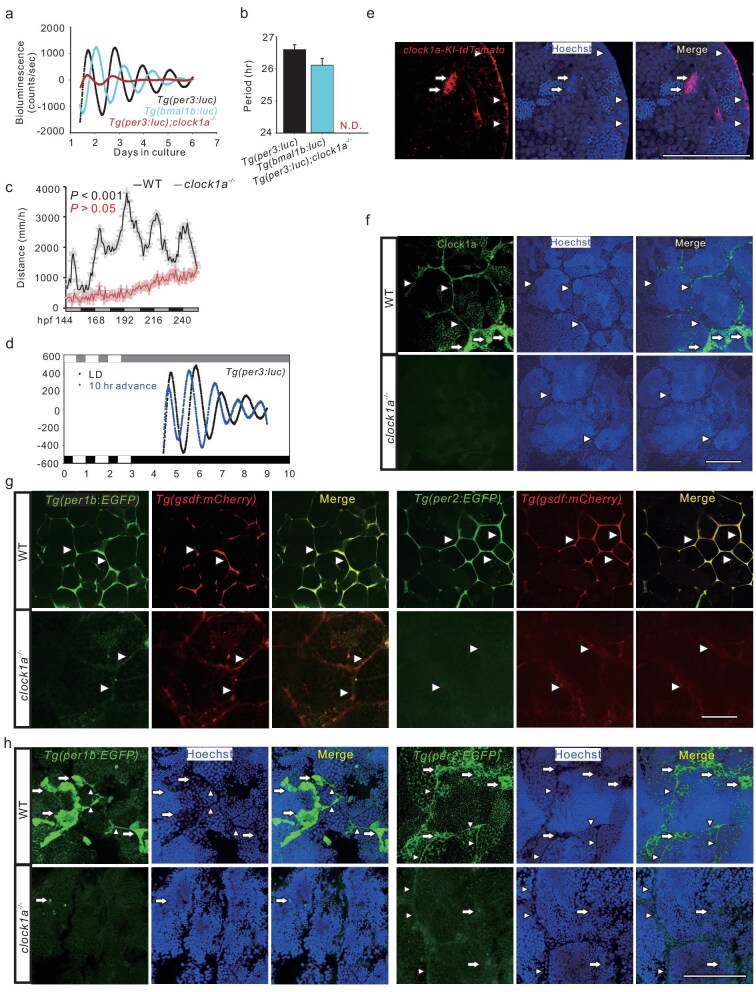
The zebrafish testis clock ticks in a cell-specific manner. (a and b) Bioluminescence recordings (a) and their periods (b) of the testis cultured *ex vivo* from *Tg(per3: luc)* (black), *Tg(per3: luc); clock1a^−/−^* (red) and *Tg(bmal1b: luc)* (cyan), detrended by 24-hour moving average (*n* = 10). (c) Locomotor rhythm analysis of 5- to 9-dpf (days postfertilization) WT (black) and *clock1a*^−/−^ (red) zebrafish larvae under DD condition (*n* = 24). (d) Detrended bioluminescence of *Tg(per3: luc)* testis recordings under 10-hour advanced conditions after the culture *ex vivo* for 3 days under normal LD (*n* = 4). (e) Confocal images of the testis from *clock1a-KI-tdTomato* zebrafish (*n* = 3). (f) Confocal images of IHC staining in *clock1a*^−/−^ and WT control testes with a mouse CLOCK antibody (*n* = 6). (g and h) Expression of *per1b* and *per2* in the Sertoli cells (g) and spermatogonia (h), and their downregulation in the *clock1a*^−/−^ Sertoli cells or spermatogonia, as shown by confocal images of the testes from the *Tg(per1b: EGFP; gsdf: mCherry), Tg(per1b: EGFP; gsdf: mCherry); clock1a*^−/−^, *Tg(per2: EGFP; gsdf: mCherry*), and *Tg(per2: EGFP; gsdf: mCherry); clock1a*^−/−^ zebrafish lines (*n* = 5–14). In (e–h), all nuclei were counterstained with Hoechst 33342. Arrowheads indicate Sertoli cells, and arrows spermatogonia. Scale bars =100 μm (Fig. S2 and Movie S1).

Our transcriptome analysis of the zebrafish and mouse testes reveals more than 1000 rhythmically expressed genes, which is consistent with 1672 out of 17554 (9.52%) genes oscillating in the baboon testis [20], even although zebrafish, mouse and baboon testes share only a few rhythmically expressed genes (Fig. S1g; and Table S2). Nevertheless, ∼5%–10% of genes oscillating in the zebrafish, mouse and baboon testes are at odds with the widely held notion of testicular arrhythmicity [12–15,17]. This observation has prompted us to re-examine the testis clock activities. Using transgenic zebrafish lines *Tg(per3: luc)* [25] and *Tg*(*bmal1b: luc)* [26], we were able to observe robust circadian oscillations of both *per3* and *bmal1b*, as shown by antiphasic bioluminescence rhythmicity driven by these two circadian clock genes, in the zebrafish testis (Fig. 2a and b). To examine if the circadian expression of *per3* observed in the testis is regulated by the circadian clock, we crossed *Tg(per3: luc)* transgenic zebrafish with *clock1a^−/−^* (also known as *clocka*) [27] zebrafish (Fig. S2a-S2d) and obtained *Tg(per3: luc); clock1a^−/−^* zebrafish. Loss of Clock1a completely abolishes *per3-*driven bioluminescence rhythmicity (Fig. 2a and b), consistent with the abolished locomotor rhythmicity (Fig. 2c) and altered expression of circadian clock genes (Fig. S2e) in *clock1a^−/−^*larvae. We also investigated whether *per3-*driven bioluminescence rhythmicity is light-entrainable, a fundamental property of the circadian clock, and observed that the *per3-*driven bioluminescence rhythm in the testis was effectively phase-shifted for 10 hours following the advanced light/dark paradigm (Fig. 2d).

To investigate exactly which tissues/cells of the testis the circadian clock genes are expressed in, we first generated a *clock1a* knock-in zebrafish expressing *tdTomato* (Fig. S2k-S2n), as previously described [28]. The Clock1a-driven *tdTomato* signals are observed primarily in Sertoli cells and spermatogonia (Fig. 2e). We then examined the Clock1a protein expression with immunohistochemistry (IHC) in wild-type (WT) and *clock1a^−/−^* testes. Clock1a is also expressed in Sertoli cells and spermatogonia, which is lost in the *clock1a^−/−^* testis (Fig. 2f). Further, we generated transgenic zebrafish lines *Tg(per1b : EGFP)* and *Tg(per2 : EGFP)*, wherein EGFP is driven by the promoters of circadian clock genes *per1b* or *per2* (Fig. S2f and S2g). We crossed these *Tg(per1b : EGFP)* and *Tg(per2 : EGFP)* transgenic zebrafish lines with *clock1a^−/−^* zebrafish and obtained *Tg(per1b : EGFP); clock1a^−/−^* and *Tg(per2 : EGFP); clock1a^−/−^* zebrafish lines, respectively. The 3D volume renderings of testes from these zebrafish lines showed that these two circadian clock genes are highly expressed in the inter-cyst areas of the WT testis but are nearly lost in those of the *clock1a^−/−^* testis (Movie S1). We then crossed *Tg(per1b : EGFP); clock1a^−/−^* and *Tg(per2 : EGFP); clock1a^−/−^* zebrafish lines with Sertoli cell-specific transgenic zebrafish *Tg(gsdf : mCherry)* [29] and obtained *Tg(per1b : EGFP; gsdf : mCherry); clock1a^−/−^* and *Tg(per2 : EGFP; gsdf : mCherry); clock1a^−/−^* zebrafish lines, respectively. Clearly, both *per1b* and *per2* are highly expressed in Sertoli cells, as shown by overlapping EGFP and mCherry signals in Sertoli cells of the testes of these two zebrafish lines, which are markedly downregulated in the *clock1a^−/−^* Sertoli cells (Fig. 2g). Moreover, both *per1b* and *per2* are also expressed in spermatogonial cells of WT fish, but are dramatically reduced in those of the *clock1a^−/−^* spermatogonial cells (Fig. 2h). Intriguingly, the circadian clock proteins Per1b and Bmal1b are also expressed in Sertoli cells and spermatogonial cells in either the WT or *clock1a^−/−^* testes (Fig. S2i). Together, these results suggest that the circadian clock operates in Sertoli cells and spermatogonial cells in a cell-specific manner, likely contributing to the cellular functions in these cell types.

### Zebrafish Sertoli cells display rhythmicity

**Figure 3.**
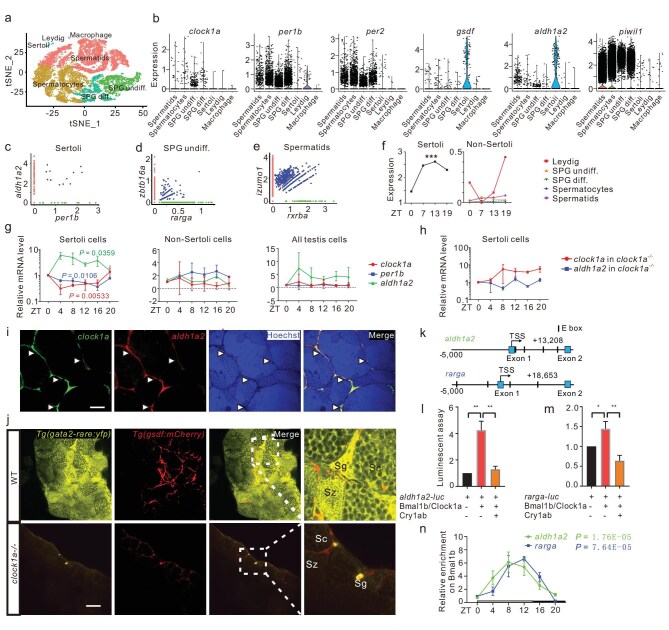
Circadian regulation of RA signaling in zebrafish. (a) scRNA-seq analysis identifies seven zebrafish testicular clusters (*n* = ∼32 000). (b) Expression of circadian clock genes and testicular genes in the testicular cell clusters. (c–e) Single-cell co-expression (blue dots) of *aldh1a2* (red dots) with *per1b* (green dots) in Sertoli cells (c), *ztbt16a* (red dots) with *rarga* (green dots) in SPG undiff. (d), and *izumol* (red dots) with *rxrba* (green dots) in spermatids (e). (f) Plot of the rhythmic expression of *aldh1a2* in Sertoli cells and its arrhythmic expression in non-Sertoli cells at four timepoints of a day, revealed by scRNA-seq. (g and h) qRT-PCR analyses of *clock1a* (red), *per1b* (blue), and *aldh1a2* (green) with RNAs from FACS-selected Sertoli cells, non-Sertoli cells, mixed cells from testicular cells of *Tg(gsdf : mCherry)* (g) and *Tg(gsdf : mCherry); clock1a^−/−^* (h) (*n* = 3 × 3). (i) Co-localization of *clock1a* (green) and *aldh1a2* (red) in Sertoli cells, as shown by double FISH (*n* = 3 × 4). All nuclei were counterstained with Hoechst 33342. Arrowheads indicate Sertoli cells. Scale bar, 50 μm. (j) Confocal images of the testes from *Tg(RARE-gata2a : NLS-EYFP; gsdf : mCherry)* and *Tg(RARE-gata2a : NLS-EYFP; gsdf : mCherry); clock1a*^−/−^ zebrafish lines (*n* = 5–7). Sg, spermatogonia; Sc, spermatocyte; Sz, spermatozoon. Scale bar, 50 μm. (k) Schematic diagrams of the *aldh1a2* and *rarga* loci harboring E-boxes. (l, m), Luciferase report assays of the *aldh1a2* (l) and *rarga* regulatory fragments (m) (*n* = 3). (n) Quantitative ChIP assays (*n* = 2). *P < 0.05; **P < 0.01 (Fig. S3).

We conducted single-cell RNA sequencing (scRNA-seq) analysis of adult zebrafish testes and identified seven major testicular cell populations/clusters, including undifferentiated spermatogonia (SPG), differentiated SPG, meiotic spermatocytes, post-meiotic spermatids, microphage, Leydig cells and Sertoli cells (Fig. 3a, Fig. S3a-S3c; and Table S3). Although the testicular somatic Sertoli cells are underrepresented in the testicular cell populations, as shown in both cell numbers acquired and gene numbers identified (Fig. 3a, Figs. S3c-S3d), the scRNA-seq analysis still detected the expression of circadian clock genes, including *clock1a, per1b* and *per2*, and Sertoli cell marker genes *gsdf* and *aldh1a2* (ENSDARG00000053493) [30] in the Sertoli cells (Fig. 3b, Fig. S3d); and the expression of circadian clock genes in both undifferentiated and differentiated SPG (Fig. 3b, Fig. S3d), consistent with what we observed in transgenic zebrafish (Fig. 2g) and IHC staining (Fig. S2i). However, the scRNA-seq analysis showed a very low level of *clock1a* at the single-cell level (Fig. 3b), implicating a gating role of Clock1a as the positive factor in the zebrafish circadian regulatory loop.

Previous studies have not yet demonstrated the rhythmic expression of circadian clock genes in the testes of zebrafish and rodents [12–15]. All of these studies used the RNAs lumped together from all the cell types from the testes [12–15], which could have swamped rhythmic gene expression in a relatively small group of a particular cell type in the testis. Our time-series scRNA-seq analysis showed rhythmic expression of RA biosynthesis gene *aldh1a2* in Sertoli cells (Fig. 3f), implicating that circadian clock genes and circadian clock-controlled genes could oscillate in a cell-specific manner. To further examine this possibility, we employed fluorescence-activated cell sorting (FACS) to select mCherry-positive Sertoli cells, mCherry-negative non-Sertoli cells, and mixed cells from disassociated testicular cells from *Tg(gsdf : mCherry)* zebrafish [29] (Fig. S3e) and were able to estimate that the number of Sertoli cells is ∼2%–3% of the total testicular cells in a 3-month-old adult male zebrafish (Figs. S3f-S3g), which was further supported by the even lower rate of Sertoli cells out of the total testicular cells, determined by the scRNA-seq analysis (Fig. S3c). We then examined the expression of *clock1a, per1b* and *aldh1a2* in Sertoli cells, non-Sertoli cells and the mixed cells from the whole testis, respectively. Remarkably, qRT-PCR showed that all three genes are rhythmically expressed only in Sertoli cells, but not in the non-Sertoli cells or the mixed cells from the whole testis (Fig. 3g). The rhythmicity of *clock1a* and *aldh1a2* disappeared in *clock1a^−/−^* Sertoli cells (Fig. 3h), while the expression level of *per1b* was too low to be detected in *clock1a^−/−^*Sertoli cells. In addition, to examine whether spermatogonial cells exhibit rhythmicity, we crossed *Tg(piwil1 : mCherry)* (*piwil1*, a spermatogonial marker) [31] zebrafish with *Tg(per3 : luc)* zebrafish or *Tg(per3 : luc); clock1a^−/−^* zebrafish, and generated *Tg(per3 : luc; piwil1 : mCherry)* and *Tg(per3 : luc; piwil1 : mCherry); clock1a^−/−^* zebrafish lines, respectively. Bioluminescence analysis of these FACS-selected spermatogonia-related cell types, including spermatogonial cells and non-spermatogonial cells from the testes of *clock1a^−/−^* and WT zebrafish (Figs. S3h-S3i), showed that none of them displays rhythmicity (Fig. S3j). Hence, only Sertoli cells exhibit rhythmicity in the zebrafish testis.

### Circadian regulation of RA signaling in zebrafish

Our transcriptome analysis reveals that several genes involved in RA signaling are rhythmically expressed and peak in the evening (Fig. 1k and l), thereby suggesting that RA signaling is under circadian regulation in the zebrafish. Our scRNA-seq analysis provided evidence for single-cell co-expression of *per1b* with *aldh1a2* in Sertoli cells (Fig. 3c). In particular, double fluorescent *in situ* hybridization (FISH) shows co-localization of *clock1a* with *aldh1a2* in Sertoli cells (Fig. 3i).

In mammals, RA biosynthesis is catalyzed by the rate-limiting enzyme ALDH1A1/ALDH1A2 (Aldehyde dehydrogenase 1 family, member A1/A2) primarily in Sertoli cells, and then RA is diffused to spermatogonia and other parts of the testis, exerting its roles via RA receptors [32]. To examine which part of the testis RA is enriched and how the loss of Clock1a affects RA synthesis and distribution in the zebrafish testis, we crossed RA sensor *Tg(RARE-gata2a : NLS-EYFP)* zebrafish [33] with *Tg(gsdf : mCherry)* zebrafish to obtain *Tg(RARE-gata2a : NLS-EYFP; gsdf : mCherry)* zebrafish, which were then crossed with *clock1a^−/−^* zebrafish to generate *Tg(RARE-gata2a : NLS-EYFP; gsdf : mCherry); clock1a^−/−^* zebrafish. Intriguingly, RA is highly concentrated in both spermatogonia and spermatozoa, but is lowly concentrated in spermatocytes, which are all notably reduced in those of the *clock1a^−/−^* mutant testes (Fig. 3j). Thus, RA synthesized in Sertoli cells is diffused to spermatogonia and spermatozoa where it presumably exerts its effects in zebrafish paracrinally as in mice [32].

Scrutinization of the *aldh1a2* and *retinoic acid receptor gamma a* (*rarga*) (ENSDARG00000034117) loci revealed an E-box enhancer in their first intron, respectively (Fig. 3k). We generated DNA constructs wherein the luciferase reporter gene is driven by the E-box–containing *aldh1a2* or *rarga* regulatory fragment. Luciferase reporter assays showed that transcriptional activities of these E-box–containing *aldh1a2* and *rarga* regulatory fragments are activated by Bmal1b and Clock1a but repressed by Cry1ab, respectively (Fig. 3l and m). Moreover, Bmal1b rhythmically binds to the E-box in the first intron of *aldh1a2* and *rarga* (Fig. 3n), respectively. Together, these results indicate that the circadian clock regulates RA signaling in the zebrafish testis.

### Genetic disruption of Clock1a results in arrested spermatogonial differentiation and reduced fertilization in zebrafish

**Figure 4.**
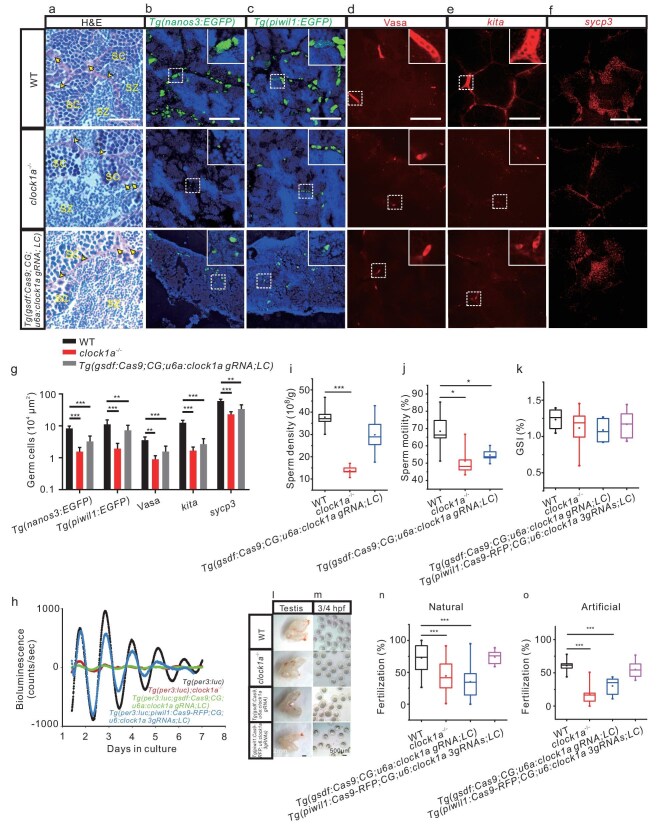
Spermatogenesis and fertilization defects in *clock1a^−/−^* and Sertoli cell *clock1a* mutant zebrafish. (a–f) Images of the H&E staining (a), fluorescent *Tg(nanos3 : EGFP)* (b) and *Tg(piwil1 : EGFP)* (c), IHC with a Vasa antibody (d), FISH with *kita* probe (e) and *sycp3* probe (f) of WT control, *clock1a*^−/−^, and Sertoli cell *clock1a* mutant testes (*n* = 9–20). (g) Quantification of the images in (b–f) (*n* = 6–16). (h) Detrended bioluminescence recordings of the testes *ex vivo* from *Tg(per3 : luc)* (black), *Tg(per3 : luc); clock1a*^−/−^ (red), *Tg(per3 : luc; gsdf : Cas9; CG; u6a : clock1a gRNA; LC)* (green), and *Tg(per3 : luc; piwil1 : Cas9-RFP; CG; u6 : clock1a 3gRNAs; LC)* (cyan) zebrafish lines (*n* = 3). (i and j) Sperm density (i) and motility (j) of WT control, *clock1a*^−/−^, and Sertoli cell *clock1a* mutant zebrafish (*n* = 5). (k–m) GSI (k), morphology (l), and images of fertilization at 3/4 hours postfertilization (HPF) (m) of WT control, *clock1a*^−/−^, Sertoli cell *clock1a* mutant, and spermatogonia *clock1a* mutant testes (*n* = 6–10). Scale bar, 500 μm. (n and o) Fertilization rates by pairwise crosses of WT control, *clock1a*^−/−^, Sertoli cell *clock1a* mutant, and spermatogonia *clock1a* mutant males with WT control females by natural crossings (n) (*n* = 10–24) or IVF (o) (*n* = 8). (a) Scale bar, 33 μm. SC, spermatocyte; SZ, spermatozoon. (b–f) Scale bar, 100 μm. (b, c) All nuclei were counterstained with Hoechst 33342. Arrowheads indicate Sertoli cells and arrows spermatogonia. *P < 0.05; **P < 0.01; ***P < 0.001 (Fig. S4 and Movie S2).

Because RA signaling is necessary for spermatogenesis [32], we hypothesized that loss of Clock1a results in dysregulated RA signaling, which in turn leads to reproductive defects. We employed hematoxylin and eosin (H&E) staining, transgenic zebrafish lines, IHC and FISH to examine the testicular cell types and the expression of germ cell markers in the testes of *clock1a^−/−^*, Sertoli cell-specific *clock1a* mutant (Figs. S4a-S4h) and WT zebrafish lines. We generated *clock1a* gRNA-expressing zebrafish lines *Tg(u6a : clock1a gRNA; LC)* with one gRNA target site (Fig. S4a) and *Tg(u6a : clock1a 3gRNAs; LC)* with three gRNA target sites (Figs. S4j-S4k), and a Sertoli cell *Cas9*-expressing zebrafish line *Tg(gsdf : Cas9; CG)* (Fig. S4b), and crossed them to obtain the Sertoli cell-specific *clock1a* mutant lines *Tg(gsdf : Cas9; CG; u6a : clock1a gRNA; LC)* and *Tg(gsdf : Cas9; CG;u6a : clock1a 3gRNAs; LC)*, as previously described [34] (Figs. S4a-S4h). While circadian clock proteins Per1b and Bmal1b are expressed in the Sertoli cell *clock1a* mutant testis (Fig. S2i), Clock1a protein is significantly downregulated in it (Fig. S2j). Importantly, disruption of *clock1a* specifically in Sertoli cells abolishes *per3-*driven bioluminescence rhythmicity in the testis as in the *clock1a^−/−^* testis (Fig. 4h). We crossed the Sertoli cell-specific *clock1a* mutant zebrafish *Tg(gsdf : Cas9; CG; u6a : clock1a gRNA; LC)* with *Tg(RARE-gata2a : NLS-EYFP; gsdf : mCherry)* zebrafish to obtain the *Tg(RARE-gata2a : NLS-EYFP; gsdf : mCherry; gsdf : Cas9; CG; u6a : clock1a gRNA; LC)* zebrafish line. Indeed, RA production is also significantly downregulated in the Sertoli cell-specific *clock1a* mutant testis (Fig. S3k).

H&E staining showed that the germ cells are significantly reduced in the *clock1a^−/−^* and Sertoli cell *clock1a* mutant testes, while the testicular cystic structures remain unchanged (Fig. 4a). Using *Tg(piwil1; EGFP)* [31] and *Tg(nanos3 : EGFP)* transgenic zebrafish lines, we observed that *piwil1*-positive cells and *nanos3*- (an early spermatogonial marker) positive cells are significantly reduced in the testes of *clock1a^−/−^* and Sertoli cell *clock1a* mutant zebrafish (Fig. 4b and c, and g). Vasa-positive early germ cells are markedly decreased in the testes of *clock1a^−/−^* and Sertoli cell *clock1a* mutant fish (Fig. 4d and g), as shown by IHC. FISH showed that *kita-*positive cells are significantly reduced in the testes of *clock1a^−/−^* and Sertoli cell *clock1a* mutant fish (Fig. 4e and g), consistent with the reduced *piwil1*-positive spermatogonial cells in the testes of the *clock1a^−/−^* and Sertoli cell *clock1a* mutant zebrafish (Fig. 4c and g); while *sycp3*- (a meiotic spermatocyte marker) [35] positive cells are also reduced in the testes of *clock1a^−/−^* and Sertoli cell *clock1a* mutant zebrafish (Fig. 4f and g). To investigate the possible effect of the circadian clock in spermatogonia, we generated a spermatogonia-specific *Cas9*-expressing zebrafish line *Tg(piwil1 : Cas9-RFP; CG)* (Fig. S4l), and crossed it with the *clock1a* gRNA-expressing zebrafish line *Tg(u6 : clock1a 3gRNAs; LC)* (Figs. S4l-S4k) to obtain the spermatogonia-specific *clock1a* mutant line *Tg(piwil1 : Cas9-RFP; CG; u6 : clock1a 3gRNAs; LC)* (Figs. S4k-S4q). We crossed the spermatogonia-specific *clock1a* mutant line with *Tg(per3 : luc)* and obtained a *Tg(per3 : luc; piwil1 : Cas9-RFP; CG; u6 : clock1a 3gRNAs; LC)* line. Unlike the arrhythmicity of the testis of Sertoli cell-specific *clock1a* mutant fish, the spermatogonia-specific *clock1a* mutant testis maintains rhythmicity (Fig. 4h), and the spermatogenesis appears to be not affected in spermatogonia-specific *clock1a* mutant testis (Figs. S4r-S4s). Hence, the loss of Clock1a globally or disruption of Clock1a Sertoli cell specifically results in arrested spermatogonial differentiation.

We then investigated whether loss of Clock1a affects fertilization. Compared with WT control zebrafish, both sperm production and sperm mobility are significantly reduced in *clock1a^−/−^* zebrafish, while only sperm mobility is decreased in Sertoli cell-specific *clock1a* mutant zebrafish (Fig. 4i and j; and Movie S2). However, it appears that the general size and morphology (Fig. 4m) of the testes are not altered in *clock1a^−/−^*, Sertoli cell-specific *clock1a* or spermatogonia-specific *clock1a* mutant zebrafish, as shown by the Gonadal somatic index (GSI) (Fig. 4k). Through crossing WT females with *clock1a^−/−^* males, Sertoli cell-specific *clock1a* mutant males, spermatogonia-specific *clock1a* mutant males, or WT males, respectively, we found that *clock1a^−/^*or Sertoli cell-specific *clock1a* mutant males, but not spermatogonia-specific *clock1a* mutant males, display significantly reduced fertilization rates (Fig. 4n and o), which is further supported by artificial *in vitro* fertilization (IVF) with the equivalent dose of sperms from the *clock1a^−/−^*, Sertoli cell-specific *clock1a* mutant, spermatogonia-specific *clock1a* mutant, and WT male zebrafish (Fig. 4o). Because loss of Clock1a seems to affect sperm motility without altering sperm morphology (Fig. 4m, Fig. S4i), the reduced fertilization rates observed in the *clock1a^−/−^*or Sertoli cell-specific *clock1a* mutants could result from the altered sperm quality rather than from the reduced quantity of sperms. Moreover, no defects in the fertilization rate were observed in the spermatogonia-specific *clock1a* mutant zebrafish (Fig. 4n and o), combined with its rhythmicity (Fig. 4h) and normalcy in the spermatogenesis (Figs. S4r-S4s), implicating no direct circadian involvement in spermatogonia, even although circadian clock genes are expressed in spermatogonia (Figs 2e and f, h, 3b, Figs. S2i-S2j, S3d). Our results suggest that loss of Clock1a globally or disruption of Clock1a Sertoli cell specifically leads to downregulated RA, arrested spermatogonial differentiation and reduced fertilization in zebrafish.

### The circadian clock-controlled RA signaling regulates *zbtb16a* and *izumo1* in zebrafish

**Figure 5.**
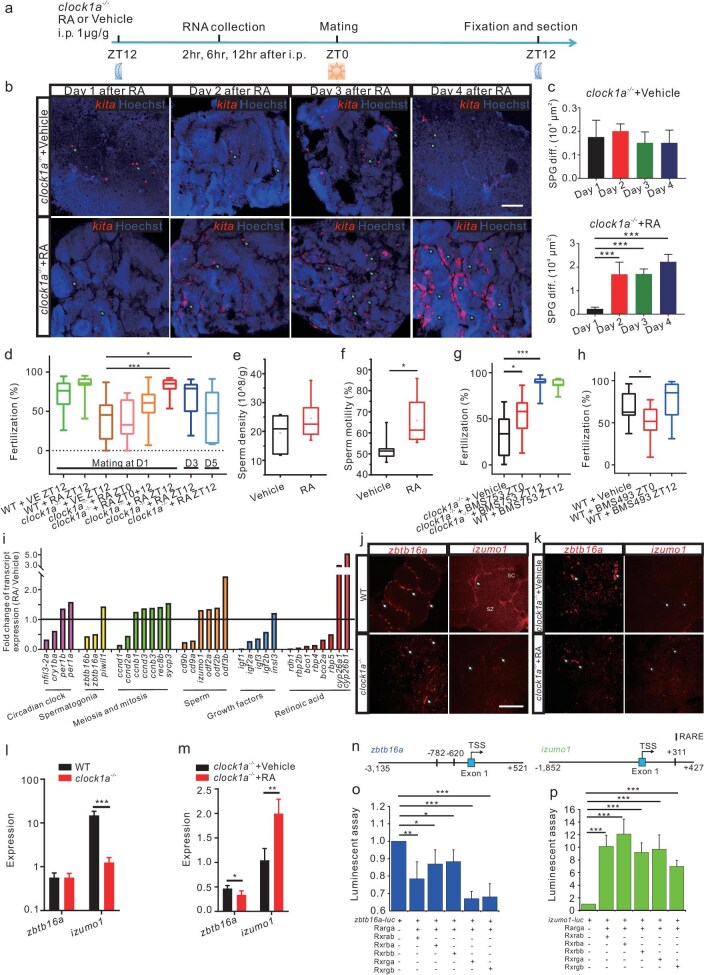
atRA treatment rescues the *clock1a*^−/−^ spermatogenesis and fertilization defects via *zbtb16a* and *izumo1* in zebrafish. (a) Schedules of the experiments. (b) FISH images of the WT and *clock1a*^−/−^ testis sections with the *kita* probe at days 1 to 4 following the atRA or vehicle treatment *i.p.* at ZT12 (*n* = 3 × 5). The signals were superimposed with the Hoechst33423 nuclear counterstain. Scale bar, 100 μm. (c) Quantification of *kita*-positive cells in b (*n* = 4). (d) Fertilization rates by pairwise crosses of WT males or *clock1a*^−/−^ males with WT females 1, 3, and 5 days after atRA or vehicle treatment at ZT0 or ZT12 (*n* = 9–18). (e and f) Sperm density (e) and motility (f) of the *clock1a*^−/−^ mutant males after atRA or vehicle treatment (*n* = 6). (g and h) Fertilization rates by pairwise crosses of WT females with *clock1a*^−/−^ males or WT males treated with BMS753 (g), and WT females with WT males treated with BMS493 (h) at ZT0 or ZT12 (*n* = 10–18). (i) The ratios of gene expression levels in the *clock1a*^−/−^ testis 2 hours after atRA treatment to those after vehicle treatment (*n* = 3). (j) FISH images of WT and *clock1a*^−/−^ testes using *zbtb16a* or *izumo1* probe (*n* = 4). Scale bar, 100 μm. (k) FISH images of *clock1a*^−/−^ testes treated with RA or vehicle using *zbtb16a* or *izumo1* probe (*n* = 4). (l and m) Quantification of *zbtb16a*-positive and *izumo1*-positive cells in (j and k) (*n* = 4). (n) Schematic diagrams of the RARE motifs-containing *izumo1* and *zbtb16a* promoters. (o and p) Luciferase reporter assays of *izumo1* (o) and *zbtb16a* (p) promoters (*n* = 3). (b, j and k) Arrows indicate spermatogonia. Sc, spermatocyte; Sz, spermatozoon. *P < 0.05; **P < 0.01;***P < 0.001 (Fig. S5).

To search for the downstream factors underlying the role of the circadian clock-controlled RA signaling in spermatogenesis and fertilization, we performed all-trans retinoic acid (atRA) and vehicle administration to the *clock1a^−/−^* and WT male zebrafish intraperitoneally (*i.p.*) and examined the expression of spermatogenesis marker genes in the testis and fertilization following atRA or vehicle administration (Fig. 5a). As WT males produce sufficient levels of RA required for spermatogenesis and fertilization, neither differences in the *kita-*expressing differentiated spermatogonia and the *sycp3*-expressing spermatocytes nor differences in fertilization rates (Fig. 5d) were observed between WT males injected with atRA and those injected with the vehicle. By contrast, from day 2 after the atRA treatment, the *kita-*expressing differentiated spermatogonia increased significantly in the *clock1a^−/−^* testes, while the *clock1a^−/−^* testes treated with the vehicle still displayed arrested spermatogenesis (Fig. 5b and c), indicating that atRA treatment effectively rescued defective spermatogonial differentiation in *clock1a^−/−^* males. However, the *sycp3*-expressing spermatocytes seemed not to be altered by the atRA treatment (Figs. S5a-S5b). Further, atRA treatment also rescued the fertilization defects in *clock1a^−/−^* in a time-of-day-specific manner (Fig. 5d), i.e. the atRA administration was most effective only at ZT12, while the *clock1a^−/−^* males injected with atRA at ZT0 still showed the reduced fertilization rate (Fig. 5d). The rescuing effect of atRA was reduced continuously from day 3 up to day 5 following the single-dose atRA injection, indicative of the transient effect of atRA on fertilization (Fig. 5d). Intriguingly, atRA treatment enhanced the sperm motility but not the sperm density 1 day after the atRA injection (Fig. 5e and f). Hence, the effect of atRA on fertilization likely results from the sperm's improved fertilization capacities rather than from the amount of sperm. In addition, pharmacological experiments showed that RARα-selective agonist BMS753 [32] was able to rescue the reduced fertilization rate of *clock1a^−/−^* when administered at ZT12 rather than at ZT0, but seemed to have no effect on the fertilization rate of WT males treated at ZT12 (Fig. 5g), while pan-RAR antagonist BMS493 [32] was able to reduce the fertilization rate of WT males when treated at ZT0 (Fig. 5h), underscoring the roles of RA signaling in fertilization. Together, these results indicate that the circadian clock-driven RA signaling synchronizes spermatogonial differentiation and promotes fertilization in zebrafish.

To identify potential molecular mediators of the circadian clock-controlled RA effects in spermatogenesis and fertilization, we conducted transcriptome analysis of the *clock1a^−/−^* testes collected 2 hours after the atRA and vehicle administration at ZT12 (Fig. S5c). The atRA treatment triggered 1813 upregulated genes (Table S4) and 5034 downregulated genes in the *clock1a^−/−^* testes treated with atRA (Table S4). GO analysis of differentially expressed genes by the atRA treatment [36,37] revealed that the most significantly upregulated genes are involved in spermatogenesis, spermatid development and sperm motility (Fig. S5d), while downregulated genes are primarily involved in metabolism (Fig. S5e). As expected, atRA treatment significantly altered the expression of circadian clock genes and genes involved in RA signaling, spermatogonia and sperm functions (Fig. 5i, Table S4). Additional transcriptome analysis of atRA-treated testes in a time-series manner showed that the regulatory effect of atRA on the expression of these genes disappeared dynamically 12 or even 6 hours after the atRA treatment (Fig. S5f, and Table S4), which likely underlies the diminishing rescue effect of atRA on fertilization (Fig. 5d). Interestingly, atRA treatment significantly upregulates *izumo1* (ENSDARG00000116848) but downregulates *zbtb16a* (ENSDARG00000007184) (Fig. 5i)*. zbtb16a*, an undifferentiated spermatogonia marker, is expressed in WT testicular inter-cyst areas, but more extensively in those of the *clock1a^−/−^* testis (Fig. 5j and l), which was downregulated after atRA treatment in spermatogonia (Fig. 5i, k, and m). *izumo1* (*izumo sperm-egg fusion 1*), a fertilization-related factor [38], is significantly downregulated in the *clock1a^−/−^* mutant in comparison with wild types (Fig. 5j and l), which was upregulated after RA treatment in germ cells (Fig. 5i, k, and m). To investigate the possible roles of Zbtb16a and Izumo1 in zebrafish reproduction, we employed CRISPR-Cas9 to generate zebrafish mutants for these two genes with multiple gRNAs (Figs. S5g-S5j). We were able to examine their phenotypes at F_0_. *In situ* hybridization showed reduced spermatogonial differentiation but no alteration of meiotic spermatocytes in the *zbtb16a* mutant testis (Figs. S5k-S5l), while a reduced fertilization rate was observed in the *izumo1* mutant zebrafish (Fig. S5m), indicative of the distinct reproductive roles of these two genes in zebrafish. Furthermore, sequence interrogation found retinoic acid response element (RARE) motifs in the promoters of *izumo1* and *zbtb16a* (Fig. 5n). We isolated RARE-containing promoters of *izumo1* and *zbtb16a* by PCR and subcloned them into the luciferase reporter vector, respectively. Luciferase reporter assays showed that combinative RA receptors Rar and Rxr effectively repress *zbtb16a* (Fig. 5o) but activate *izumo1* (Fig. 5p). scRNA-seq analysis also showed the single-cell co-expression of *zbtb16a* with *rarga* in zebrafish undifferentiated spermatogonia (Fig. 3d), and *izumo1* with *rxrba* in zebrafish spermatids (Fig. 3e). Together, these results suggest that the circadian clock-controlled RA signaling exerts its effects on spermatogonial differentiation through *zbtb16a* and on fertilization via *izumo1* in zebrafish.

### The conserved role of the Sertoli cell clock in mice

**Figure 6.**
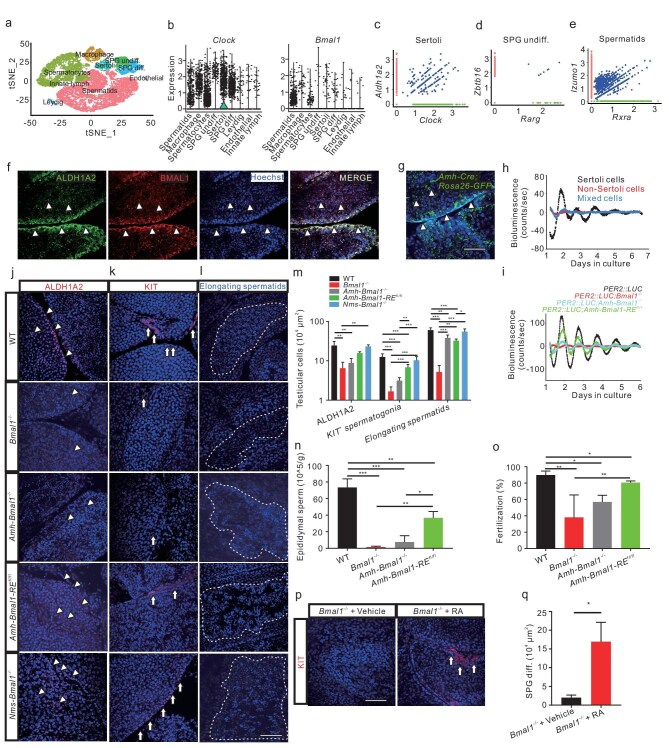
The conserved role of the Sertoli cell clock in mice. (a) scRNA-seq analysis identifies nine mouse testicular clusters (*n* = ∼35 000). (b) Expression of *Clock* and *Bmal1* in the mouse testicular cell clusters. (c–e) Single-cell co-expression (blue) of *Aldh1a2* (red) with *Clock* (green) in Sertoli cells (c), *Zbtb16* (red) with *Rarg* (green) in SPG undiff. (d), and *Izumo1* (red) with *Rxra* (green) in spermatids (e). (f) Confocal IHC image shows co-localization of ALDH1A2 (green) and BMAL1 (red) in Sertoli cells (*n* = 3 × 3). (g) Fluorescent image of EGFP-labeled Sertoli cells of *Amh-ROSA26-EGFP^fl/+^* mice (*n* = 3 × 3). (h) Bioluminescence analysis of FACS-selected Sertoli cells (black), non-Sertoli cells (red) and mixed testicular cells (blue) from *Amh-Rosa26-EGFP^fl/+^; PER2: : LUC* mouse testis (*n* = 3). (i) Bioluminescence recordings of the testes *ex vivo* from *PER2: : LUC* (black), *PER2: : LUC; Bmal1^−/−^* (red), *PER2: : LUC; Amh*-*Bmal1^−/−^* (cyan), and *PER2: : LUC; Amh*-*Bmal1-RE ^fl/fl^*(green) mice (*n* = 3–6). (j–l) Fluorescent images of WT control, *Bmal1^−/−^* KO, *Amh*-*Bmal1^−/−^, Amh*-*Bmal1-RE ^fl/fl^*, and *Nms-Bmal1^−/−^* testes with ALDH1A2 (j) and KIT (k) antibodies, and their elongating spermatids (l) (*n* = 5–10). (m) Quantification of the images in (j–l) (*n* = 3–8). (n and o) Epididymal spermatozoa (n) of WT control, *Bmal1^−/−^* KO, *Amh*-*Bmal1^−/−^*, and *Amh*-*Bmal1-RE ^fl/fl^*(*n* = 3), and fertilization rates of their sperms with WT oocytes by IVF (*n* = 3) (o). (p) IHC images of KIT in *Bmal1^−/−^* KO testes 1 day after atRA treatment at ZT12 (*n* = 4). (q) Quantification of the KIT cells in p (*n* = 4). (f to g, j to l, and p) ALL nuclei were counterstained with Hoechst 33342. Arrowheads indicate Sertoli cells and arrows spermatogonia. Scale bar, 100 μm. *P < 0.05; **P < 0.01; ***P < 0.001 (Figs S6 and S7).

We then examined the role of the circadian clock in the mouse testis. First, we re-analyzed the mouse testis scRNA-seq data published previously [39], and identified nine cell populations/clusters, including undifferentiated SPG, differentiated SPG, spermatocytes, spermatids, Sertoli cells and Leydig cells (Fig. 6a). The scRNA-seq analysis showed the expression of circadian clock genes such as *Clock* and *Bmal1* and reproduction-related genes in mouse testicular clusters (Fig. 6b, Fig. S6c). Like the low level of *clock1a* in the zebrafish testis (Fig. 3b), the scRNA-seq analysis also detected a very low level of *Bmal1* at the single-cell level (Fig. 6b), supporting the notion of the gating role of both zebrafish Clock1a and mouse BMAL1 in the circadian network. Further, the scRNA-seq analysis also detected single-cell co-expression of *Clock* with *Aldh1a1* in Sertoli cells (Fig. 6c), of *Zbtb16* with *Rarg* in undifferentiated spermatogonia (Fig. 6d), and of *Izumo1* with *Rxra* in spermatids (Fig. 6e). Further, IHC staining shows the co-localization of BMAL1 and ALDH1A2 in the mouse Sertoli (Fig. 6f).

To investigate whether mouse Sertoli cells display rhythmicity, *Rosa26*-*EGFP ^fl/+^* mice [40] were interbred with *Amh*-*P2A*-*iCre* mice to obtain Sertoli cell-specific EGFP mice, i.e. *Amh-Rosa26-EGFP ^fl/+^* (Fig. 6g), which were then interbred with the *PER2: : LUC* mice [41] to generate Sertoli cell-specific EGFP mice with *PER2: : LUC*, i.e. *Amh-Rosa26-EGFP ^fl/+^; PER2: : LUC*. Then, EGFP-positive Sertoli cells, EGFP-negative non-Sertoli cells, and mixed testicular cells were selected with FACS, cultured, and analyzed with Lumicycle, respectively. As expected, the rhythmicity of bioluminescence driven by *Per2* was observed only in Sertoli cells, not in non-Sertoli cells or mixed testicular cells (Fig. 6h), consistent with what we observed in zebrafish (Fig. 3g). Further, we were able to observe robust rhythmicity in the WT testis, which was abolished in the global *Bmal1^−/−^* KO [42] and Sertoli cell *Bmal1* KO (*Amh*-*Bmal1^−/−^*) mouse testes, but partially restored in Sertoli cell *Bmal1* RE (reconstituted) (*Amh*-*Bmal1-RE ^fl/fl^*) mouse testis (Fig. 6i). By examining ALDH1A2 expression, KIT-positive spermatogonia, and elongating spermatids in the testes of *Bmal1^−/−^*, Sertoli cell *Bmal1* KO, Sertoli cell *Bmal1* RE, SCN *Bmal1* KO, and WT control mice, we observed significant downregulation of ALDH1A2 in the *Bmal1^−/−^* and Sertoli cell *Bmal1* KO testes but its partial rescue in the Sertoli cell *Bmal1* RE testes (Fig. 6j and m), and markedly reduced KIT-positive spermatogonia and elongating spermatids in the testes of *Bmal1^−/−^* and Sertoli cell *Bmal1* KO mice, which can be restored in the testis of Sertoli cell *Bmal1* RE mice (Fig. 6k–m). Even although both the circadian rhythmicity of locomotor activity and synchrony of the circadian clock are abolished in SCN *Bmal1* KO mice [43], no alteration of ALDH1A2 expression, KIT-positive spermatogonia, elongating spermatids and litter size was observed in SCN *Bmal1* KO mice (Fig. 6j–m, Fig. S7f), indicating that the Sertoli cell clock plays a much more important role in reproduction than the SCN central clock.

We observed significant reductions in epidydimal spermatozoa (Fig. 6n) and fertilization rate (Fig. 6o) in *Bmal1^−/−^* KO and Sertoli cell *Bmal1* KO mice and significantly reduced litter size in Sertoli cell *Bmal1* KO mice (Fig. S7f), consistent with that global *Bmal1^−/−^* KO mice are infertile [44]. Intriguingly, compared with global *Bmal1^−/−^* KO and Sertoli cell *Bmal1* KO mice, Sertoli cell *Bmal1* RE mice display not only significant increases in ALDH1A2 expression (Fig. 6j and m), KIT-positive spermatogonia (Fig. 6k and m) and elongating spermatids (Fig. 6l and m), but also increased epidydimal spermatozoa and fertilization rates (Fig. 6n and o), indicating that the Sertoli cell clock plays important roles in spermatogenesis and fertilization in mice. However, the reconstitution of the Sertoli cell clock failed to restore the litter size (Fig. S7f), implicating that, in addition to spermatogenesis and fertilization, the circadian clock likely acts in other reproductive processes such as implantation and steroid hormonal production in mammals [45], which also affect the litter size and fertility. Similar to the observation in zebrafish, the spermatogonia defects in *Bmal1^−/−^* KO mice could be rescued by RA injection *i.p.* at ZT12 (Fig. 6p and q). Together, these results indicated that the mouse Sertoli cell clock plays conserved regulatory roles in spermatogenesis and fertilization, similar to the zebrafish Sertoli cell clock.

### Temporally desynchronizing the circadian clock leads to arrested spermatogonial differentiation and reduced fertilization

Our results demonstrate that genetic disruption of Clock1a/BMAL1 globally or Sertoli cell specifically causes arrested spermatogonial differentiation and reduced fertilization in both zebrafish and mice. Previous epidemiological studies showed that circadian misalignment, such as rotating or shift work, is highly associated with reduced sperm production and lower reproduction [46,47]. We hypothesize that temporal desynchronization of the circadian clock also leads to altered spermatogenesis and reduced reproduction. To examine this hypothesis, we generated a heat shock-inducible *clock1a*-overexpressing transgenic zebrafish line *Tg(hsp70l : clock1a; CG2)* (Figs. S8a-S8e). Intriguingly, in comparison with abolished locomotor rhythms in *clock1a^−/−^* zebrafish under constant darkness (DD) (Fig. 2c), heat shock 1 hour either at ZT0, when *clock1a* typically has the nadir expression level, or at ZT12, when *clock1a* commonly peaks, *Tg(hsp70l : clock1a; CG2)* larvae still maintain locomotor rhythmicity under DD and LD condition (Figs. S8f-S8i).

**Figure 7.**
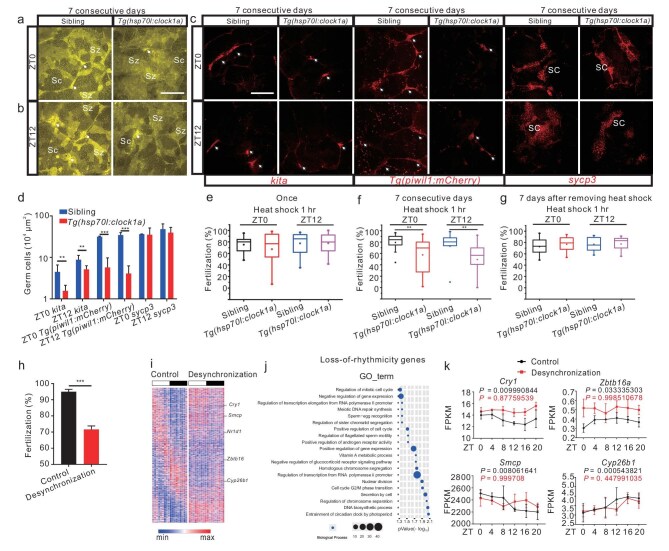
Temporally desynchronizing the circadian clock results in arrested spermatogonial differentiation and reduced fertilization in zebrafish and mice. (a and b) Confocal images of the testes from *Tg(RARE-gata2a : NLS-EYFP; hsp70l : clock1a; CG2)* and sibling males after heat shock at ZT0 (a) or ZT12 (b) for 7 consecutive days (*n* = 9–15). (c) FISH images of *Tg(hsp70l : clock1a; CG2)* and sibling male testes with *kita* or *sycp3* probe, and confocal images of *Tg(hsp70l : clock1a; CG2; piwil1 : mCherry)* and sibling male testes, after heat shock at ZT0 or ZT12 for 7 consecutive days (*n* = 3 × 5). (d) Quantification of the images in (c) (*n* = 6 −18). (e–g) Fertilization rates by pairwise crosses of WT females and *Tg(hsp70l : clock1a; CG2)* males or sibling males following heat shock just once (e) or for 7 consecutive days (f) starting at ZT0 or ZT12 or 7 days after removing the treatment from heat shock starting at ZT0 or ZT12 for 7 consecutive days (g) (*n* = 3 × 10). (h) Fertilization rates of desynchronization and control groups’ sperms with WT oocytes by IVF (*n* = 3). (i) Heatmaps of loss-of-rhythmicity genes in the desynchronization and control groups. (j) Top-20 enriched GO terms BP of the loss-of-rhythmicity genes in the desynchronization group. (k) Expression of *Cry1, Smcp, Zbtb16* and *Cyp26b1* in the control (black) and desynchronization (red) groups. (a–c), Arrows indicate spermatogonia. Sg, spermatogonia; Sc, spermatocyte; Sz, spermatozoon. Scale bar, 100 μm. **P < 0.01; ***P < 0.001 (Figs S8 and S9).

We then crossed *Tg(hsp70l : clock1a; CG2)* zebrafish with *Tg(RARE-gata2a : NLS-EYFP; gsdf : mCherry)* zebrafish and generated *Tg(RARE-gata2a : NLS-EYFP; gsdf : mCherry; hsp70l : clock1a; CG2)* zebrafish. Remarkably, RA production is significantly downregulated, and its distribution is notably disrupted by *clock1a*-overexpressing with heat shock at either ZT0 or ZT12 for 7 consecutive days (Fig. 7a and b), but seems unaltered by overexpressing *clock1a* only once (Fig. S8j). Further, RA production and distribution appear to resume as normal 7 days after terminating the temporal perturbation following heat shock at either ZT0 or ZT12 for 7 consecutive days (Fig. S8k). Spermatogonial differentiation was arrested following the 7-consecutive-day *clock1a* overexpression, but the meiotic spermatocytes and sperm production were not affected (Fig. 7c and d). Intriguingly, *clock1a* overexpression by heat shock once at either ZT0 or ZT12 appeared to have no effect on spermatogonial differentiation (Fig. S8l); and spermatogonial differentiation can resume as normal 7 days after terminating the temporal perturbation following heat shock at either ZT0 or ZT12 for 7 consecutive days (Fig. S8m). Further, following heat shock once at either ZT0 or ZT12, we examined the fertilization rates and found no significant effect of the one-time *clock1a* overexpression on fertilization (Fig. 7e). Then, we performed overexpression at ZT0 or ZT12 for 7 consecutive days and found that fertilization rates were significantly reduced (Fig. 7f), which could be recovered 7 days after terminating the heat shock treatment (Fig. 7g). Hence, chronically disrupting the circadian clock also results in downregulated RA, arrested spermatogenesis and reduced fertilization in zebrafish.

Similarly, reduced sperm count under circadian desynchronization and its recovery after terminating circadian desynchronization were recently observed in a mouse model [47]. We conducted transcriptome analysis of the mouse testes (Table S5) and also examined the fertilization rates and sperm counts, after the 1-month-long desynchronizing treatment (Fig. S9a). The epidydimal spermatozoa were reduced, as previously shown [47], and the fertilization rate was also reduced in the desynchronization group (Fig. 7h), indicating that 1-month desynchronization abolished the spermatogenesis and sperm capacity to fertilize. In transcriptome analysis, out of 1879 rhythmically expressed genes, 1473 genes were revealed to lose rhythmicity in the desynchronization group, including circadian clock genes *Cry1* and *Nr1d1* (Fig. 7i; and Table S5). The top-20 enriched GO are BP terms of these loss-of-rhythmicity genes are involved in testicular functions, such as sperm-egg recognition, sperm motility, meiotic cell cycle and circadian entrainment (Fig. 7j). Specifically, *Cyp26b1*, encoding an RA degradation enzyme, *Zbtb16*, an undifferentiated spermatogonia marker gene, and *Smcp* (Sperm mitochondria-associated cysteine-rich protein), its loss causing asthenozoospermia [48], all lost their rhythmicity in the desynchronization group (Fig. 7i–k), implicating that circadian desynchronization likely leads to dysregulated RA signaling and defects in spermatogenesis and fertilization in mice. By contrast, 1829 genes gained rhythmicity in the desynchronization group, including *Rara, Amh, Amhr2* and *Kiss1* (Figs. S9b-S9d; and Table S5). Intriguingly, there were 406 genes maintaining rhythmicity in both desynchronization and control groups, including genes functioning in spermatogenesis and fertilization (Figs. S9e-S9g, and Table S5). Notably, most of these rhythmicity-maintaining genes, loss-of-rhythmicity genes and gain-of-rhythmicity genes also display altered phases in the desynchronization group (Fig. S9h and Table S5). Together, these results indicated that circadian misalignment, such as perturbing the clockwork temporally for consecutive days or desynchronizing by continuous phase shifts, likely leads to arrested spermatogonial differentiation and reduced fertilization, providing evidence for the epidemiological observation that men with chronic circadian misalignment suffer from reduced fertility.

## DISCUSSION

Our studies demonstrate that the testis unequivocally displays robust rhythmic activities in zebrafish and mice. In particular, the testis clock ticks in a cell-specific manner : canonical circadian clock genes are not only rhythmically expressed (Fig. 3f), but also regulate RA production in Sertoli cells in zebrafish (Fig. 3i and j, and l). The circadian clock-controlled RA signaling contributes to zebrafish testicular functions in two ways; one is to synchronize spermatogonial differentiation via downregulating transcription suppressor *zbtb16a* (Fig. 5i and o); the other is to promote fertilization via upregulating sperm-egg fusion factor *izumo1* (Fig. 5i and p); and in both ways, RA synthesized in Sertoli cells is diffused to spermatogonia or spermatozoa (Fig. 3j) and exerts its paracrine roles, respectively. Moreover, the mouse Sertoli cell clock (Fig. 6h and i) appears to play a conserved role in spermatogonial differentiation (Fig. 6j–m, p and q) and fertilization (Fig. 6n and o).

Our qRT-PCR and/or bioluminescence analyses showed that canonical circadian clock genes are rhythmically expressed only in the zebrafish (Figs 3g, 4h) and mouse Sertoli cells (Fig. 6h). This testicular rhythmicity is Clock1a/BMAL1-dependent in zebrafish (Figs 2a and b, 3g and h) and mice (Fig. 6i). As Sertoli cells are only ∼2%–3% of the total testicular cells in zebrafish (Fig. S3c, S3g) and mice (Fig. S6b), examining the expression of circadian clock genes with RNAs extracted from the whole testis would mask the rhythmicity of the small-proportion Sertoli cells, which should account for why previous studies used RNAs lumped together from the whole testis failed to reveal circadian rhythmicity of the zebrafish and mouse testes [12–15]. Further, our results suggest that time-series RNA-seq is more effective in detecting rhythmically expressed genes than traditional hybridization-based Northern blotting or RNA protection assays, as it allows for estimating gene expression levels per sample.

Our studies reveal that the circadian clock directly controls RA biosynthesis in Sertoli cells, as shown by circadian regulation of *aldh1a2* (Fig. 3k–m); RA is then diffused to spermatogonia (Fig. 3j) and synchronizes its differentiation, and to spermatozoa (Fig. 3j) to capacitate sperms and, in turn, promotes fertilization. Further, we also showed that Clock1a, Per1b and Bmal1b proteins are expressed in Sertoli cells and spermatogonia (Fig. 2e–h, Figs. S2i). However, *per3-*driven bioluminescence is arrhythmic in spermatogonia labeled by *piwil1* (Fig. S3j). As *piwil1* positive cells include undifferentiated spermatogonial stem cells and differentiated spermatogonial cells [31], arrhythmicity of undifferentiated spermatogonial stem cells would be in line with the absence of circadian rhythmicity of murine and human embryonic stem cells [49]; however, our bioluminescence analysis also implied the arrhythmicity of differentiated spermatogonial cells, presumably the major portion of the *piwil1* positive cells. In particular, spermatogonia-specific *clock1a* mutant zebrafish display no defects in spermatogonia differentiation (Figs. S4r-S4s) and fertilization rate (Fig. 4n and o), implicating no direct circadian involvement in spermatogonia.

The scRNA-seq analyses of zebrafish, mouse and human testes showed that canonical circadian clock genes are expressed in Sertoli cells (Figs 3b and c, 6b–e, Figs. S3d, S6c-S6e, S6h, S6k). However, the Sertoli cells detected by the scRNA-seq analysis of zebrafish (Fig. S3c) are much fewer than those in mice (Fig. S6b) and humans (FigS6f), maybe due to the different cell dissociation methods [39,50]. Quantifying gene expression at the single-cell levels is still tricky because of the relatively low capture of cell numbers and gene numbers per cell (Figs. S3c, S6b, S6f). However, the scRNA-seq analysis provided evidence for single-cell co-expression of *per1b*/*Clock*/*CLOCK* with *aldh1a2*/*Aldh1a2*/*ALDH1A2* in Sertoli cells in zebrafish (Fig. 3c), mice (Fig. 6c) and humans (Fig. S6h), implicating that the circadian clock-controlled RA synthesis in Sertoli cells is highly conserved from zebrafish, mice to humans. Further, the scRNA-seq analysis also showed much lower levels of *clock1a/Bmal1/BMAL1* in Sertoli cells of the zebrafish (Fig. 3b), mouse (Fig. 6b) and human testes (Figs. S6e, S6k), implicating the notion of Clock1a/BMAL1 as a gating factor in the circadian network.

Genetic deletion of Clock1a leads to a complete loss of the rhythmicity of locomotor behavior and testicular rhythmicity (Fig. 2a–c), indicative of the critical role played by Clock1a in zebrafish circadian regulation and testicular functions. Zebrafish also possess *clock1b*, and *clock1a* and *clock1b* are co-orthologs of the mammalian *Clock* gene as an ancient duplicate pair [51]. Indeed, *clock1b^−/−^* zebrafish (Figs. S9i-S9j) still maintain partial locomotor rhythmicity (Fig. S9k), and importantly, loss of Clock1b barely affects fertilization (Fig. S9l), indicating that it is Clock1a that plays the primary role in the testis clock.

Disruption of Clock1a or BMAL1 either globally or Sertoli cell-specifically results in reduced germ cells in general and decreased differentiated spermatogonial cells, in particular in zebrafish (Fig. 4a–g) and mice (Fig. 6j–m). Endogenous RA is highly enriched in zebrafish spermatogonial cells (Fig. 3j), and atRA treatment can effectively rescue arrested differentiated spermatogonia of the *clock1a^−/−^* male zebrafish (Fig. 5b and c) or the *Bmal1^−/−^* KO male mouse (Fig. 6p and q), which is consistent with the role of RA in spermatogonial differentiation in mice and zebrafish [30,32,52,53]. In particular, the atRA treatment significantly downregulates undifferentiated spermatogonia marker *zbtb16a* (Fig. 5i, k, m), indicating that Zbtb16a likely mediates the role of the circadian clock-regulated RA signaling in spermatogonial differentiation. Intriguingly, the scRNA-seq analysis showed single-cell co-expression of *ztbt16a/Ztbt16/ZTBT16* with *rarga/Rarg/RARG* in undifferentiated spermatogonia of zebrafish (Fig. 3d), mice (Fig. 6d) and humans (Fig. S6i), implicating the conserved role of the circadian clock-controlled RA signaling in undifferentiated spermatogonia from zebrafish, mice, to humans. Further, endogenous RA is also highly enriched in spermatozoa/spermatids (Fig. 3j), and the atRA treatment can effectively rescue reduced fertilization rates in the *clock1a^−/−^* mutant male zebrafish (Fig. 5d), which is the first time that the role of RA in fertilization has been revealed. In particular, the atRA treatment upregulates *izumo1* (Fig. 5i, k, m), but not the sperm numbers (Fig. 5e), implicating that RA in spermatozoa/spermatids likely plays a role in capacitating sperm's ability to fertilize the egg via upregulating *izumo1.* The scRNA-seq analysis also detected single-cell co-expression of *izumo1/Izumo1/IZUMO1* with RA receptor genes *rxrba/Rxra/RXRA* in spermatids of zebrafish (Fig. 3e), mice (Fig. 6e) and humans (Fig. S6J), implicating the conserved role of the circadian clock-controlled RA signaling in fertilization from zebrafish, mice, to humans. Indeed, both network and Circos plots [54] show highly conserved ligands-receptors-mediated crosstalk among the circadian clock-regulated RA in Sertoli cells, RA receptors-expressed spermatogonia and spermatids from zebrafish (Figs. S7g-S7h), mice (Figs. S7i-S7j) and humans (Figs. S7k-S7l).

Intriguingly, reconstitution of *Bmal1* in Sertoli cells can partially rescue the defects of spermatogonial differentiation (Fig. 6k and m) and fertilization (Fig. 6o), but not fertility (Fig. S7f), suggesting that circadian clock-controlled RA signaling contributes largely to spermatogonial differentiation and fertilization, which is highly conserved in vertebrates. Numerous other reproductive processes, such as embryo implantation and steroid hormonal production [45], also affect fertility in mammals. A previous study reported oscillations of the BMAL1 protein in the mouse Leydig cells and suggested BMAL1 acts through the steroidogenic acute regulatory protein (*StAR*) gene to affect testosterone synthesis [44]. However, contradictory observations, such as the downregulation of *StAR* in the testes of *Bmal1^−/−^* and *Per2^−/−^* mice but the upregulation of *StAR* in those of *Clock^−/−^* and *Per1^−/−^* mice, cast doubt on the transcription/translation loop-based circadian regulation in the mouse Leydig cells [44].

Our inducible model showed that overexpression of *clock1a* at ZT0 or ZT12 for 7 consecutive days led to altered RA production and distribution (Fig. 7a and b), arrested spermatogonial differentiation (Fig. 7c and d) and reduced fertilization (Fig. 7f), while one-time *clock1a* overexpression appeared to have no such effects (Fig. S8j). Intriguingly, RA production and distribution (Fig. S8k), spermatogonial differentiation (Fig. S8m) and fertilization (Fig. 7g) can resume as normal 7 days after terminating temporal perturbation of *clock1a* expression; temporal perturbation of *clock1a* expression for 7 consecutive days does not affect sperm counts (Fig. S8n). Similarly, long-term desynchronization of mice also leads to lower sperm count [47] and reduced fertilization rates (Fig. 7h), and, in particular, the rhythmicity of canonical circadian clock genes and genes involved in spermatogenesis and fertilization was disputed by circadian desynchronization (Fig. 7i–k). These findings have far-reaching implications for those men in modern society who have chronically disrupted their circadian systems and manifested reduced fertility [8].

In conclusion, our studies reveal that the circadian clock located in Sertoli cells synchronizes spermatogonial differentiation and promotes fertilization by controlling RA biosynthesis and RA receptors. Circadian disruption, either disruption of *clock1a/Bmal1* globally or Sertoli cell specifically, or temporally perturbing *clock1a* expression or desynchronizing, all results in arrested spermatogonial differentiation and reduced fertilization in zebrafish and mice (Fig. S10). These findings pinpoint robust testicular rhythmicity and circadian roles in male fertility. Hence, we have discovered a new peripheral clock responsible for reproductive loss in modern society's huge shift-work population, revealing a novel underlying mechanism involved in clock-controlled RA signaling.

## MATERIALS AND METHODS

Detailed materials and methods are available in the supplementary data.

## Supplementary Material

nwae456_Supplemental_Files
