# Supplementary material for: The circadian clock orchestrates spermatogonial differentiation and fertilization by regulating retinoic acid signaling in vertebrates
Source: Natl Sci Rev. 2024 Dec 11;12(3):nwae456. doi: 10.1093/nsr/nwae456 (PMC11884735; doi:10.1093/nsr/nwae456)
Supplement: nwae456_Supplemental_Files [file nwae456_supplemental_files.zip › Supplementary data.pdf]

Supplementary data for

The circadian clock orchestrates spermatogonial differentiation and fertilization by regulating retinoic acid (RA) signaling in vertebrates

Taole Liu [1,2], Wei He [1,2], Zhaomin Zhong [1,2], Chenchen Lu[1,2], Lianxin Wu[1,2], Ziming Wang[1,2], William Kojo Smith[1,2], Quan Shi[1,2], Qiaoming Long [3], Han Wang [1,2]*

[1] Center for Circadian Clocks, Soochow University, Suzhou 215123, Jiangsu, China

[2] School of Basic Medical Sciences, Suzhou Medical College, Soochow University, Suzhou 215123, Jiangsu, China

[3] Cam-Su Genomic Resource Center, Soochow University, Suzhou, 215123, China

* Corresponding: wanghan@suda.edu.cn or han.wang88@gmail.com (H. W.)

This PDF file includes:

MATERIALS AND METHODS, Supplementary Figs. S1 to S10, captions of Supplementary Tables S1 to S5, and Supplementary Movies S1 to S2, and Supplementary Tables S6 to S7.

MATERIALS AND METHODS

Zebrafish husbandry

Zebrafish (*Danio rerio*) wild-type AB strain, transgenic and mutant lines are maintained at 28.5 ^{0}C on a 14 hr/10 hr light/dark cycle at the Soochow University Zebrafish Facility according to standard protocols [1]. All zebrafish protocols were approved by the Soochow University Animal Ethics and Use Committee (SUDA20230811A01).

Zebrafish mutant and transgenic lines

The following zebrafish strains are used in this study: *Tg(per3:luc)* [2], *Tg(bmal1b:luc)* [3], *Tg(piwil1:EGFP)* [4], *Tg(piwil1:mCherry)*, *Tg(gsdf:mCherry)*[5], and *Tg(RARE-gata2a:NLS-EYFP)* [6]. We also generated the following zebrafish lines *clock1a*$^{-/-}$*, Tg(hsp70l:clock1a;CG2), clock1b*$^{-/-}$, *ztbt16a* mutant, *izumo1* mutant, *Tg(per1b:EGFP)*, *Tg(per2:EGFP)*, *clock1a knock-in*, *Tg(gsdf:Cas9;CG2)*, *Tg(u6a:clock1a gRNA;LC)*, *Tg(u6:clock1a 3gRNAs;LC)*, *Tg(gsdf:Cas9;CG2;u6a:clock1a gRNA;LC)*, *Tg(piwil1:Cas9-RFP;CG2)*, *Tg(piwil1:Cas9-RFP;CG2;u6:clock1a 3gRNAs;LC), and Tg(nanos3:EGFP)* (Supplementary Table S7).

Mouse care and behavior assays

The mouse strains are raised in LD (12 h light/12 h dark) conditions at the Soochow University SPF animal facility credited by AAALAC (the Association for Assessment and Accreditation of Laboratory Animal Care). The following mouse stains are used in the study: C57BL/6J, *Bmal1*$^{-/-}$ KO [7], *Bmal1-loxp* [8], *Rosa26-GFP*$^{fl/+}$ [9], *PER2::LUC* [10], and *Nms*-Cre [11]. We also generated *Bmal1-RE* and *Amh-P2A-iCre* mice (Supplementary Table S7). All mouse protocols were approved by the Soochow University Animal Care and Use Committee (SUDA20230811A01). Wheel-running activity assays were conducted, and the behavioral data were analyzed with ClockLab (Actimetrics) software as previously described [12].

Generation of mutant, transgenic, and knock-in zebrafish lines

1. *clock1a*$^{-/-}$ *zebrafish*. The *clock1a*$^{-/-}$ mutant with 7-bp deletion was generated with CRISPR-Cas9 as previously described [13]. A gRNA target site with a BslI restriction site in Exon 3 of *clock1a* was selected (Supplementary Fig. S2a); the *in vitro* synthesized gRNA and *Cas9* mRNA were co-injected into one-cell zebrafish embryos; the microinjected embryos were raised to adulthood; the mutant lines were screened with PCR and fin-clipped DNAs, and confirmed by DNA sequencing analysis; and the 7-bp deletion mutant with only 101 amino acids was used for the study (Figs. S2a-S2d). **2**. *Tg(per1b:EGFP)* and *Tg(per2:EGFP) zebrafish*. These two transgenic zebrafish lines were generated as previously described [14]. The 1,953-bp *per1b* promoter, PCR amplified with primers 5' AAGCCTGAGGACACATTGCT-3' and 5'-TTCTGTTGAACGACAAGAC-3'; and the 1,821-bp *per2* promoter, PCR amplified with primers primer 5'-TTCCCAGTGCTTAGTGGCAG-3' and 5'-TAACACCGCTGCCCTGGATC-3', were cloned into the *Tol2*-containing vector *pT2-EGFP*; the resultant DNA constructs p*per1-EGFP* and p*per2-EGFP* were microinjected into one-cell embryos with *Tol2* transposase, respectively; the microinjected embryos were raised to adulthood; and the transgenic lines were screened with PCR and fin-clipped DNAs, and confirmed by DNA sequencing (Supplementary Figs. S2f-S2g). **3**. *clock1a knock-in zebrafish*. We generated a *clock1a knock-in* (KI) zebrafish line as previously described [15]. The gRNA target site was selected in Intron 17 of the *clock1a* locus. The fragment of the partial Intron 17 sequence after the 3' *clock1a* gRNA target site, and the remaining *clock1a* cDNA sequence at the beginning of Exon 18 were amplified by PCR, respectively. Then, the FoRe vector was digested with KpnI and AvrII, and the two amplified fragments were subcloned into the FoRe vector, to generate the *clock1a* FoRe plasmid, using the ClonExpress MultiS One Step Cloning Kit (Vazyme Biotech). To enhance their efficiencies, the *clock1a* gRNA and *hEMXI* gRNA were modified with 2'-O-methyl-3'-phosphorothioate (MS) at Tsingke Biotech

(tsingke.com) as previously described [16]. *Cas9* mRNA and *clock1a* gRNA were microinjected into one-cell zebrafish embryos. 24 hours after the microinjection, the efficiency was determined by enzymatic digestion of the PCR-amplified fragment (Supplementary Fig. S2l). Then *Cas9* mRNA, *clock1a* gRNA, *hEMXI* gRNA, and the *clock1a* FoRe plasmid were microinjected into one-cell zebrafish embryos. The microinjected embryos were grown to maturity, and then they were crossed with wild-type zebrafish to produce F_1 embryos. Junction PCRs with primers at the 5' and 3' ends were used to screen F_1 embryos carrying the *clock1a* KI FoRe (Supplementary Fig. S2n). After these *clock1a* KI FoRe F_1 embryos were grown to maturity, they were incrossed to produce F_2 embryos. These F_2 zebrafish were driven to homozygosity and verified with PCR amplification and DNA sequencing analyses. **4.** *Sertoli cell-specific clock1a mutant and spermatogonia-specific clock1a mutant zebrafish*. These *clock1a* conditional zebrafish lines were generated as previously described [17]. In short, the 2 046-bp *gsdf* promoter, PCR amplified with primers 5'-ACCCAGGCCACCATTACAGAACT-3' and 5'-ATGTCTGTGGATTCAGGAGCGT-3'; and the 4.8-kb *piwil1* promoter [18] were subcloned into the p5E vector; the *Cas9* cDNA was subcloned into the pME vector; and then LR reactions were used to assemble the *gsdf* or *piwil1* promoter and *Cas9* into the final vector. The *clock1a* gRNA site (the same one in Exon 3 of *clock1a*) (Supplementary Fig. S4a) and three gRNA sites (the same one in Exon 3, and two additional gRNA sites: one in Exon 4, and the other in Exon 7) (Supplementary Figs. S4j-S4l) were subcloned into the *cryaa*-CFP vector with the *u6a* promoter, respectively. The resultant DNA constructs p*u6a-clock1a* gRNA-LC (Supplementary Fig. S4a), p*gsdf-Cas9-CG2* (Ext Supplementary Fig. S4b), p*piwil-Cas9-P2A-RFP-CG2* (Supplementary Fig. S4k), and p*u6-clock1a* 3gRNAs-LC (Supplementary Fig. S4l), were microinjected into one-cell embryos with *Tol2* transposase, respectively; the microinjected embryos were raised to adulthood; and the heritable transgenic lines were screened with PCR and fin-clipped DNAs, and confirmed by sequencing analysis (Supplementary Figs. S4c-S4h, S4m-S4q). *Tg(gsdf:Cas9;CG2)* zebrafish were crossed with *Tg(u6a:clock1a gRNA;LC)* zebrafish or *Tg(u6:clock1a 3 gRNA;LC)* zebrafish to obtain the Sertoli cell *clock1a* mutant lines *Tg(gsdf:Cas9;CG2;u6a:clock1a gRNA;LC)* and *Tg(gsdf:Cas9;CG2;u6:clock1a 3gRNAs;LC)*, respectively. Similarly, *Tg(piwil1:Cas9-RFP;CG2)* zebrafish was crossed with *Tg(u6:clock1a 3gRNAs;LC)* zebrafish to generate the spermatogonia *clock1a* mutant line *Tg(piwil1:Cas9-RFP;CG2;u6:clock1a-3gRNAs;LC)*. **5**. *ztbt16a and izumo1 mutants*. These zebrafish mutants were generated with multiple gRNAs (at least three). Individual gRNA and *Cas9* mRNA were first microinjected into one-cell embryos, and their efficiencies were estimated by the TIDE (https://tide.nki.nl/) analysis of the PCR products of the targeted fragment [19]. At least three gRNAs with relatively higher efficiencies were simultaneously microinjected with *Cas9* mRNA into one-cell embryos; the microinjected embryos were raised to adulthood; and the *ztbt16a* and *izumo1* mutant lines were screened with PCR and fin-clipped DNAs, confirmed by sequencing analysis (Supplementary Figs. S5g-S5j), and characterized at F_0 (Supplementary Figs. S5k-S5m). **6**. *The clock1a-overexpressing transgenic zebrafish*. This transgenic line was generated as previously described [20]. Briefly, a Gateway destination vector containing a heat shock-inducible promoter 5' to attR, an SV40 polyadenylation signal 3' to attR2, and the entire cassette flanked by *Tol2* transposase arms; and LR reaction (Invitrogen) was used to link *clock1a* ORF into the destination vector (Supplementary Fig. S7a). An injection mix (50 ng/μl plasmid and 150 ng/μl *Tol2* transposase mRNA) was injected into one-cell embryos; the microinjected embryos were raised to adulthood; and the transgenic lines were screened with PCR and fin-clipped DNAs and confirmed by sequencing analysis (Supplementary Figs. S7b-S7e). **7**. *Tg(nanos3:EGFP)*. This transgenic line was generated using the same *nanos3* promoter and EGFP as previously described [21].

Generation of *Bmal1-RE* (reconstituted) and *Amh-P2A-iCre* mice

1. *The Bmal1-RE mouse*. The *Bmal1*-TerminationFL mouse was generated using C57BL/6J mice as previously described [22]. Briefly, a termination cassette containing a splice acceptor (SA) (GSG: GGAAGCGGA), a mCherry reporter gene, a poly-A tail, and an *FRT*-flanked neomycin resistance cassette, flanked by *loxP* sites, was introduced

into Intron 7 of the *Bmal1/Arntl1* gene via CRISPR-Cas9-mediated targeted DNA insertion (gRNA site, TGTGGGACCCGAGTGCTCTA) (Supplementary Fig. S7a). The *FRT*-flanked neomycin resistance cassette was used for clonal selection and removed by *FLP*-mediated recombination after successful germline transmission (Supplementary Fig. S7b). *Cre*-mediated recombination excises the *loxP*-flanked termination cassette and the mCherry reporter, thereby reconstituting the wild-type *Bmal1/Arntl1* allele (Supplementary Fig. S6c). **2**. *Amh-P2A-iCre mouse*. The *Amh*-specific *iCre* mouse was generated with C57BL/6J mice by a CRISPR-Cas9-mediated knock-in strategy. The mouse *Amh* gene (*Amh*-201, ENSMUST00000036016.5) has five exons, with the TGA stop codon in Exon 5. *Cas9* mRNA, sgRNA (gRNA site, AGGAGGAGGGCGGGCGTCAC), and *iCre* donor were co-injected into one-cell zygotes (Supplementary Fig. S7d). The sgRNA directed *Cas9* endonuclease cleavage near stop coding (TGA) of the *Amh* gene and created a DSB (double-strand break) (Supplementary Fig. S7d). Such breaks were repaired and resulted in P2A-iCre before the stop codon (TGA) of the *Amh* gene by homologous recombination. The pups were genotyped by PCR, and confirmed by DNA sequencing analysis. The *Bmal1*-TerminationFL mice were mated with *Amh-P2A-iCre* mice to produce Sertoli cell-specific *Bmal1* reconstituted mice, *i.e.*, Sertoli cell *Bmal1* RE (*Amh-Bmal1-RE*$^{fl/fl}$). In addition, *Bmal1*$^{fl/fl}$ mice were mated with *Amh-P2A-iCre* mice or *Nms-iCre* mice to generate Sertoli cell-specific *Bmal1* KO mice, *i.e.*, Sertoli cell *Bmal1* KO (*Amh-Bmal1*$^{-/-}$) and SCN-specific *Bmal1* KO mice, *i.e.*, SCN *Bmal1* KO (*Nms-Bmal1*$^{-/-}$), respectively. Sertoli cell *Bmal1* RE (*Amh-Bmal1-RE*$^{fl/fl}$) mice, Sertoli cell *Bmal1* KO (*Amh-Bmal1*$^{-/-}$) mice, and SCN *Bmal1* KO (*Nms-Bmal1*$^{-/-}$) mice were PCR identified and confirmed by DNA sequencing analysis (Supplementary Figs. S6c, S6e).

Zebrafish behavioral analysis

Zebrafish behavioral assays were performed as described previously [3]. Briefly, on the fourth dpf (day postfertilization), larvae were placed individually into wells of a 48-well plate. Locomotor activities of the larvae were monitored for seven consecutive days under the LD or DD conditions using an automated video-tracking system (Videotrack, ViewPoint Life Sciences; or DanioVision Tracking System, Noldus Information Technology), and the movement of each larva was recorded and analyzed using Zebralab3.10 software (ViewPoint Life Sciences) or Ethovision 10.0 software (Noldus Information Technology). 48-well plates were placed inside the Zebrabox or DanioVision Observation Chamber, where continuous infrared light was illuminated and white light was illuminated from 09:00 to 23:00. Instruments were placed in the chamber to maintain a constant temperature of 28.5°C. For the DD assay, the mutant and wild-type embryos were kept under LD conditions for the first 3-day development. On the fourth dpf, the mutant and wild-type larvae were transferred to the observation chamber of the DanioVision Tracking System (Noldus Information Technology) or Videotrack (ViewPoint Life Sciences). The light was set at 300 lux during the daytime at 4 dpf, 0 lux during nighttime at 4 dpf, and then at 30 lux for all subsequent days for the DD condition starting from 09:00 at 5 dpf. Activities were measured from days 5 to 10 postfertilization, and swimming distances of the larvae were recorded in 10 min time bins. Each behavioral assay was performed at least three times.

RNA-seq-based transcriptome analysis and functional annotation

We conducted transcriptome analyses for five sets of the testicular samples. **1**. *The first set* was zebrafish time-series testis samples each with two duplicates collected for two consecutive days with a 4-hour interval under LD condition, each sample with mixed testes from three 3- to 4-month-old adult zebrafishes. **2**. *The second set* was mouse time-series testis samples each with two duplicates collected for two consecutive days with a 4-hour interval under LD condition, each sample from one mouse aged 10 weeks. **3**. *The third* set was the samples each with triplicate independent samples collected from *clock1a*$^{-/-}$ testis two hours after the atRA or vehicle treatment, each sample with mixed testes from three adult zebrafishes treated. **4**. *The fourth set* was testis samples collected from *clock1a*$^{-/-}$ testis two, six, and 12 hours after the atRA or vehicle treatment, each sample with mixed testes from three adult zebrafishes

treated. **5**. *The fifth set* was mouse time-series- testis samples each with triplicate samples collected for one day with a 4-hour interval under LD condition, from the desynchronized and control groups, each sample from one mouse aged 2-month. The one-month-long desynchronizing treatment was performed as previously described [23]. RNA-seq-based transcriptome analysis was conducted as described previously [24]. Total RNAs from each sample were extracted with TRIzol (Invitrogen). The RNA-sequencing of these testicular total RNAs was performed as follows. **1**. *Library construction for sequencing*. A total amount of 3 μg RNA per sample was used for constructing sequencing libraries, which were generated using NEBNext®Ultra™ RNA Library Prep Kit (NEB, USA) following the manufacturer's instructions. **2**. *Clustering and sequencing*. Clustering of the index-coded samples was performed on a cBot Cluster Generation System using TruSeq PE Cluster Kit (Illumina, PE-401-3001) according to the manufacturer's instructions. After clustering, the library preparations were sequenced on an Illumina Hiseq X 10 platform. **3**. *Quality control*. We calculated the Q20, Q30, GC-content, and duplication data, and then generated the raw reads. All the following analyses were based on clean data with high quality. **4**. *Transcriptome assembly*. Transcriptome assembly was performed as below. These clean reads were mapped to the zebrafish genome (GRCz11) or the mouse genome (GRCm38.p6). Only reads with a perfect match or one mismatch were further analyzed and annotated based on the reference genome. Hisat2 tool software was used to map. These raw data were deposited to NCBI with SRA accession numbers PRJNA579855 (zebrafish) and PRJNA789131 (mouse). **5**. *Gene functional annotation*. The Gene Ontology (GO) enrichment analysis was implemented by the GOseq R packages-based Wallonia noncentral hyper-geometric distribution [22]. For the Kyoto Encyclopedia of Genes and Genomes (KEGG) analysis (http://www.genome.jp/kegg/), we used KOBAS software to test the statistical enrichment of genes in KEGG pathways to predict and classify functions of the assembled sequences (https://www.genome.jp/kegg/). Gene expression levels were estimated by FPKM for each RNA sample. **6**. *Principal component analysis (PCA)*. PCA was carried out with Matlab. The first component captures most of the variance in the data. The compressed data in this study is visualized and analyzed using the first and second principal components. **7**. *DAVID ontology*. Transcripts found to be significantly regulated by RA were examined using the DAVID ontology software to identify significantly regulated pathways using Gene Ontology (GO) terms through the functional annotation chart function [25]. These significant GO terms were then clustered and visualized using the enrichment map Metascape tool [26]. A subset of enriched terms was selected and rendered as a network plot, where terms with a similarity > 0.3 are connected by edges. The network is visualized using Metascape [26], where each node represents an enriched term and is colored first by its cluster ID.

Single-cell RNA sequencing analysis of zebrafish, mouse, and human testes

The testes were collected from two 3-month-old zebrafish males each at ZT1, ZT7, ZT 13, and ZT 19, respectively, and the single-cell suspension was prepared according to the single-cell suspension protocol [27]. Then, the single-cell suspension was loaded into a Chromium microfluidic chip and barcoded with a 10× Chromium Controller (10X Genomics). RNAs from the barcoded cells were reverse-transcribed, and sequencing libraries were constructed with reagents from a Chromium Single Cell reagent kit (10X Genomics) according to the manufacturer's instructions. Sequencing was performed with Illumina NovaSeq 6000 according to the manufacturer's instructions (Illumina). Raw reads were demultiplexed and mapped to the reference genome (Zebrafish GRCz11) by 10X Genomics Cell Ranger pipeline using default parameters. A total of 32,000 cells were captured and analyzed from these four testis samples (8,000 cells per sample). All subsequent single-cell analyses were performed using Cell Ranger and Seurat packages. In brief, for each gene and each cell barcode (filtered by CellRanger), unique molecule identifiers (UMIs) were counted to construct digital expression matrices. Secondary filtration by Seurat: A gene with expression in more than three cells was considered as being expressed, and each cell was required to have at least 50 expressed genes. Certain foreign cells were filtered out by higher expression of mtRNA. The raw data and processed data have been uploaded

to NCBI (GSE2026024). In the scRNA-seq analysis of zebrafish testis, feature-barcode matrices were run, and the Seurat package was used to normalize the dimensionality reduction, clustering, and differential expression of the data. For clustering, highly variable genes were selected, and the principal components based on those genes were used to draw a graph with a resolution of 1.0. The zebrafish testis cells are divided into seven clusters, including undifferentiated spermatogonia (SPG Undiff.), differentiated spermatogonia (SPG Diff.), spermatocytes, spermatids (including spermatozoa), macrophage, Leydig cells, and Sertoli cells (Fig. 3A). In the reanalysis of the mouse [27] and human [28] testis scRNA-seq data, a gene with expression in more than three cells was considered as being expressed, and each cell was required to have at least 100 expressed genes. The mouse testis cells are divided into nine clusters, including undifferentiated spermatogonia (SPG Undiff.), differentiated spermatogonia (SPG Diff.), spermatocytes, spermatids, Sertoli cell, Leydig cell, endothelial cell, innate lymph, and macrophage (Fig. 6A), while the human testis cells are also divided into nine clusters: undifferentiated spermatogonia (SPG Undiff.), differentiated spermatogonia (SPG Diff.), spermatocytes, spermatids, Sertoli cell, Leydig cell, endothelial cell, myoid, and macrophage (Supplementary Fig. S6D). iTALK [29] was used to specify and visualize the complexity of RA signaling-mediated communication among testis clusters, including Sertoli cells, spermatogonia, and spermatids. Since iTalk is built on capturing highly abundant ligand-receptor gene pairs, we first set the ligand-receptor list as the limit-enzyme of RA signaling, the representative of the ligands, and the RAR/RXR receptors. The highly expressed ligand-receptor pairs were calculated to find top 50 percent highly expressed genes between cell clusters. The network and Circos plots [30] are used to visualize the crosstalk between Sertoli cells, spermatogonia, and spermatids mediated by RA signaling.

Fluorescence-activated cell sorting (FACS) and qRT-PCR

The testes from *Tg(gsdf:mCherry)*, *Tg(gsdf:mCherry);clock1a*$^{-/-}$, *Tg(gsdf:mCherry;per3:luc)*, *Tg(gsdf:mCherry;per3:luc);clock1a*$^{-/-}$, *Tg(piwil1:mCherry;per3:luc)*, and *Tg(piwil1:mCherry;per3:luc);clock1a*$^{-/-}$ male zebrafish at age of three months with approximately 0.3-g body weight, and *Amh-Rosa26-EGFP*$^{fl/+}$*;PER2::LUC* male mice were collected and minced in the medium L15 or DMEM in the presence of 4% papin as previously reported [27]. After 30-min digestion, pipette 15 times and filter by 40 μm filter. The cell suspension was transferred immediately onto MOFLO ASTRIOS Flow Cytometry System (Beckman Coulter). The mCherry- or EGFP- positive, -negative, or all mixed cells were collected simultaneously for 200,000 cells in TRK lysis buffer, and total RNAs were extracted from these sorted cells immediately by MicroElute ® Total RNA Kit (Omega, R6831). cDNAs were synthesized with Oligo (dT) for mRNA. Then qRT-PCR was performed in an ABI StepOne-Plus instrument with the SYBR Green detection system (Takara) and a thermal profile of 40 cycles of 95 ^{0}C for 10 s and 60 ^{0}C for 30 s. Each qRT-PCR was performed with at least three different biological samples, each in triplicates (*n* = 3 X 3). All results were normalized to the expression level of the housekeeping gene *β-actin*. Part of the FACS-selected cells were used for LumiCycle-based bioluminescence assays as described below.

Bioluminescence analysis of zebrafish and mouse testis, as well as FACS-selected cells by LumiCycle

LumiCycle-based bioluminescence assays were done on testes from adult zebrafish *Tg(per3:luc)*, *Tg(per3:luc);clock1a*$^{-/-}$, *Tg(bmal1b:luc)*, *Tg(per3:luc;gsdf:Cas9;CG2;u6a:clock1a gRNA;LC)*, and *Tg(per3:luc;piwil1:Cas9;CG2;u6:clock1a 3gRNA;LC)* zebrafish lines, adult *PER2::LUC*, *PER2::LUC;Bmal1*$^{-/-}$, *PER2::LUC;Amh-Cre;Bmal1*$^{fl/fl}$, *PER2::LUC;Amh-Rosa26-GFP*$^{fl/+}$ mouse strains, as well as specific cell types selected from the testes of adult *Tg(piwil1:mCherry;per3:luc)* and *Tg(piwil1:mCherry;per3:luc);clock1a*$^{-/-}$ fish lines, and adult *Amh-Rosa26-EGFP*$^{fl/+}$*;PER2::LUC* mice by FACS (see **Fluorescence-activated cell sorting (FACS) and qRT-PCR**). Animals were anesthetized, sacrificed, and then the testes were dissected out, immediately placed in cold Hank's balanced saline solution (HBSS, Invitrogen) on ice, and then transferred on a Millicell semi-permeable membrane (PICMORG50, Millipore) and cultured in 35 mm dishes (Nunc) with 1 ml L15 medium (for zebrafish) or

DMEM medium (for mouse) supplemented with 10% BSA (Sigma) and 0.1 mM luciferin (Promega). Dishes were sealed with plastic membranes and placed into the LumiCycle machine (LumiCycle, Actimetrics) at 28°C (for zebrafish) or 35°C (for mice). Light emission from cultured tissues was measured immediately and without interruption for seven days. Each plate was counted for 1 min and 48 sec for every 15 min. For the LumiCycle assay using adult testes, at least three testes for each transgenic zebrafish line or three mouse individuals were used, while for the LumiCycle assay using FACS-selected cells, 1 to 3 sets of approximately 200,000 cells collected from 1-5 adult zebrafish or mouse testes for each cell type were used. Cell cultures were used until they reached 70% confluence. Each LumiCycle assay was performed at least three times (n = 3). Waveforms of rhythmic bioluminescence emission were analyzed using the LumiCycle software package.

Hematoxylin and eosin (H&E) staining and *in situ* hybridization

Zebrafish testicular samples were fixed in 4% paraformaldehyde at 4^0C for 24 hours and embedded in paraffin. Transverse, 6-µm-thick, histological sections were stained with hematoxylin and eosin. *In situ* hybridization assays using digoxigenin- or fluorescein-labeled probes to detect *aldh1a2*, *kita*, *sycp3*, *zbtb16a*, *izumo1*, and *clock1a* expression were performed on the testicular sections or whole-mount testes as described previously [31]. The DNA fragments for synthesizing *clock1a*, *kita, zbtb16a*, and *izumo1* RNA probes were PCR amplified with testicular cDNAs and primers listed in Supplementary Table S6, respectively. DNA fragments for synthesizing *aldh1a2* and *sycp3* RNA probes were kindly provided by John Postlethwait [31]. *clock1a*, *zbtb16a*, *izumo1*, *kita*, and *sycp3* RNA probes were *in vitro* transcribed with a digoxigenin-labeling mixture (Roche 11277073910). For double-color *in situ* hybridization, the *aldh1a2* RNA probe was labeled with Fluorescein RNA Labeling Mixture (Roche 11685619910). Dual fluorescence *in situ* hybridization was conducted with the whole-mount testis as previously described [5]. Briefly, the fixed tissue with 4%PFA was permeabilized by protein K for 30 minutes. The antisense RNA probes of *clock1a* and *aldh1a2* were incubated with samples at 65°C for 16 hours, and the colors were developed by Alexa Fluor 488 and Alexa Fluor 555 (Invitrogen B40912 and B40923). Each *in situ* hybridization was conducted at least three times, each with at least three testes (n = 3 X 3).

Antibodies and immunohistochemistry

Zebrafish Bmal1b and Per1b polyclonal antibodies were reported previously [32]. The Bmal1b antibody was used in chromatin immunoprecipitation (ChIP) assays, while both antibodies were used for immunofluorescence staining experiments. The mouse CLOCK antibody (Santa Cruz, sc-271603) was used to detect Clock1a in zebrafish and was effective in Western blotting (Supplementary Fig. S2H). Immunohistochemistry was performed with the whole-mount testis. Briefly, the fixed tissue with 4%PFA was permeabilized by protein K for 30 minutes. Zebrafish Bmal1b and Per1b antibodies (1:100), and a Vasa antibody (a kind gift of Bruce Draper) [5] (1;1000), or mouse CLOCK, BMAL1 (MBL, D335-3), ALDH1A2 (Abcam, ab75674), KIT (Cell Signaling, D13A2) antibodies were incubated with samples at 37°C overnight, and the color stains were developed by Alexa Fluor 488 and Alexa Fluor 555 (Invitrogen B40912 and B40923). Each antibody staining experiment was performed at least three times, each with at least three testes (n = 3 X 3).

Imaging acquisition and analysis

The fluorescent images from EGFP or fluorescent *in situ* hybridization or antibody staining were acquired by confocal microscopy systems (Leica, SP8 or Nikon, A1 MP) with 405, 488, and 543 nm lasers. All nuclei are counterstained with Hoechst 33342 to allow accurate staging based on nuclear morphology in the zebrafish or mouse testis. The intensities of positive cells with distinct colors were determined by the ImageJ software (NIH). 3D video of *Tg(per1b:EGFP)*, *Tg(per2:EGFP)*, *Tg(per1b:EGFP);clock1a*$^{-/-}$ and *Tg(per2:EGFP);clock1a*$^{-/-}$ were acquired by

scanning every 5 μm, followed by volume rendering with Nikon software. Images of *Tg(RARE-gata2a:NLS-EYFP;gsdf:mCherry)*, *Tg(RARE-gata2a:NLS-EYFP;gsdf:mCherry;clock1a*$^{-/-}$, *Tg(RARE-gata2a:NLS-EYFP;gsdf:mCherry;gsdf:Cas9;CG;u6a:clock1a gRNA;LC)*, and *Tg(RARE-gata2a:NLS-EYFP;gsdf:mCherry;hsp70l:clock1a;CG2)* zebrafish lines, as well as *Amh-Rosa26-GFP*$^{fl/+}$ transgenic mouse were acquired by scanning, and analyzed with Nikon software.

Scanning electron microscopy (SEM)

Zebrafish sperm samples were fixed in 2.5 % glutaric dialdehyde (Sigma, G5882) buffer and embedded for 24 hours, and their SEM images were acquired with scanning electron microscopes (HITACHI S-4700 Field Emission Scanning Electron Microscope or ZEISS EVO18 Scanning Electron Microscope) at the Soochow University Electron Microscopy Core Facility.

Cell transfection and luciferase reporter assays

We conducted cell transfection and luciferase reporter assays as previously described [3]. A 200-bp *aldh1a2* fragment containing the E-box enhancer in the first intron, PCR amplified with primers 5'-TTTAACGCATCACCAGAGCC-3' and 5'-GGTGCGCACACACACAAAC-3', and a 200-bp *rarga* fragment containing the E-box enhancer in the first introns, PCR amplified with primers 5'-ATATTTATCAGCTTTGATTC-3' and 5'-CGATCGAGTCTGAAACCACC-3', a 2 279-bp *izumo1* promoter, PCR amplified with primers 5' TGCTGATTGGCTCACAGATAGAACC-3' and 5'-CCCTATCGACGGATAGGGTAGGTAT-3', and a 3,716-bp *zbtb16a* promoter, PCR amplified with primers 5'-CCTAAATCTAGCACTAAACCCG-3' and 5'- GTGTTGCGCGTTTGGCTTCC-3', were cloned into the pGL4.17 vector (Promega), respectively. Full-length cDNAs of *clock1a*, *bmal1b*, and *cry1ab* were reported previously [33], and full-length cDNAs of *rarab*, *rxrab*, *rxrba*, *rxrbb*, *rxrga*, and *rxrgb* were kindly provided by Qingshun Zhao. Human Embryonic Kidney (HEK) 293T cells were cultured in DMEM containing 10 % serum and Penicillin-Streptomycin in a 24-well plate. Transfection was done with Lipofectamine 2000 (Invitrogen, 11668027) according to the manufacturer's instructions. Luciferase reporter assays were performed with a Dual-Report assay system (Promega, E1980). 100 ng each of p*aldh1a2-luc*, p*rarga-luc*, p*izumo1-luc*, p*zbtb16a-luc*, *clock1a*, *bmal1b*, *cry1ab,* p*rarab*, p*rxrab*, p*rxrba*, p*rxrbb*, p*rxrga*, and p*rxrgb* were used. Three independent experiments were conducted for each assay (n = 3).

Pharmaceutical treatments

All-trans-retinoic acid (RA) (Sigma R2625) was dissolved at 0.5 mg/mL in 1% ethanol/corn oil and kept at 4 °C under agitation until administered by an *i.p.* (Intraperitoneal) injection (1 μg/g body weight to rescue arrested spermatogenesis and/or reduced fertilization) on either zebrafish *clock1a*$^{-/-}$ or mouse *Bmal1*$^{-/-}$. BMS493 (Sigma B6688) and BMS753 (Sigma SML0286) were diluted in DMSO at 50 and 2.5 mg/mL, kept at −20 °C, and diluted in corn oil right before being injected intraperitoneally (10 μg/g body weight for BMS493 and 5 μg/g body weight for BMS753).

Computer-aided sperm analysis

The zebrafish testes were freshly collected and weighted, then put in Hank's balanced saline solution (HBSS, Invitrogen). Right after tenderly squeezing the testes to help release sperms, 10-μl system water was mixed with 10-μl sperm suspension, which was examined immediately by Beion automatic sperm quality analyzer (Beion, S3-3). The time-lapse movies of sperms were first acquired by the Beion equipment, and then the sperm density and motility were determined by the Beion S3-3 software.

Temporally desynchronized mouse model

The 8-week-old male mice (C57BL/6J) were separated into two groups, the control group in normal LD (12 h light/12 h dark) condition and the desynchronized group as the paradigm as previously described [23]. In brief, for the desynchronized group, mice were placed in alternating light-cycle conditions with a 12-hr light/dark shift every six days for four times. Activity monitoring of animals was recorded using running wheels, and actogram data analysis was performed using Clocklab. After the last shift, all groups of mice remained in the same photoperiod for at least seven days before collecting testicular samples.

Mouse fertilization assays with *in Vitro* fertilization

We selected 3 to 4-week C57BL/6J female mice to achieve superovulation by intraperitoneal injection of hormones each injected with 5 IU pregnant horse serum gonadotropin (PMSG), 48 hours later injected with 5 IU human chorionic gonadotropin (hCG)]. Approximately 13-15 hours after the injection of hCG, the female mice were humanely sacrificed, and the lower abdominal cavity was opened and the tubal enlargement was cut, and the cumulus oocyte complexes (COCs) were dissected out for *in vitro* fertilization (IVF). Then we cut the cauda epididymis and part of the vas deferens from healthy three-month adult male mice. After removing blood vessels and adipose tissue, we cut several incisions to obtain mature sperm. After swimming for 10 minutes, we removed tissue fragments and let sperms swim out. The sperms were ready for *in vitro* fertilization one hour after capacitation (this step was performed prior to obtaining the cumulus oocyte complex). After the sperm capacitation was completed, inject an appropriate dose of viable sperms from the edge of the TYH (Toyoda, Yokoyama and Hosi) [34] droplet into the droplet of cumulus-oocyte complexes (COCs) collected before fertilization for 4-6 hours. After the fertilization completed, the cumulus cells were digested and atomized due to the active sperm acrosome reaction, and the fertilized eggs with good morphology were selected from the fertilized droplets and transferred into new HTF (Human tubal fluid) droplets to continue culturing to obtain early embryos after dividing into two cells.

Chromatin immunoprecipitation (ChIP) assays

Chromatin immunoprecipitation (ChIP) assays were performed with Chromatin Immunoprecipitation Assay Kit (Millipore 17-295) as described previously [3]. For the Bmal1b ChIP assay, the testes were collected at a 4-hour interval. The samples were cross-linked in 2 % formaldehyde for 10 min and then fractionated with protein A Agarose. Chromatin lysates were immunoprecipitated with the Bmal1b antibody or negative control mouse IgG (Invitrogen, 10400C) in the presence of Protein A Agarose. Then DNA was purified using a purification kit (PROMEGA, D2500) and subsequently analyzed by qPCR using primers flanking the predicted binding sites in the enhancer regions. Each ChIP assay was conducted at least three times ($n = 3$).

Rhythmicity analyses

Average FPKM values of two duplicates of the zebrafish or mouse testicular 12-time points or of triplicates of the mouse testicular six-time points in control or desynchronization groups were analyzed by meta2d, a function of the R package MetaCycle, to evaluate periodicity in the RNA-seq data [35]. Briefly, meta2d incorporates ARSER, JTK_CYCLE, and Lomb-Scargle and implements N-version programming concepts using a suite of algorithms and integrating their results (P and Q values, period, phase, and amplitude). Genes were considered to be rhythmically expressed when the integrated $P < 0.05$. We used BioDare2 (https://biodare2.ed.ac.uk/) for Period Analysis [36]. MFourFit was used, and phases were calculated on the average of the two acrophases in 2 days of transcriptome analysis. To analyze the rhythmicity of annotated groups of genes, we used Phase Set Enrichment Analysis (PSEA) software [37]. Files containing the list of cycling genes relative to a given GO (Gene Ontology) annotation and their peak phases of expression were input into PSEA software. PSEA evaluated each selected gene set for evidence of

temporally coordinated transcription and provided summary statistics. To examine the difference in RNA-seq data between the control and desynchronization groups, the Limorhyde method (R package, Limorhyde, and Limorhyde2) was used, with the Limma method for linearizing the rhythmic model [38].

Statistical analysis

Values are means ± s.d. of the indicated number of measurements. Statistical significance was determined using a two-tailed unpaired Student's *t*-test, one-way ANOVA, or two-way ANOVA with a significance of $P < 0.05$.

Supplementary Fig. S1 to S10

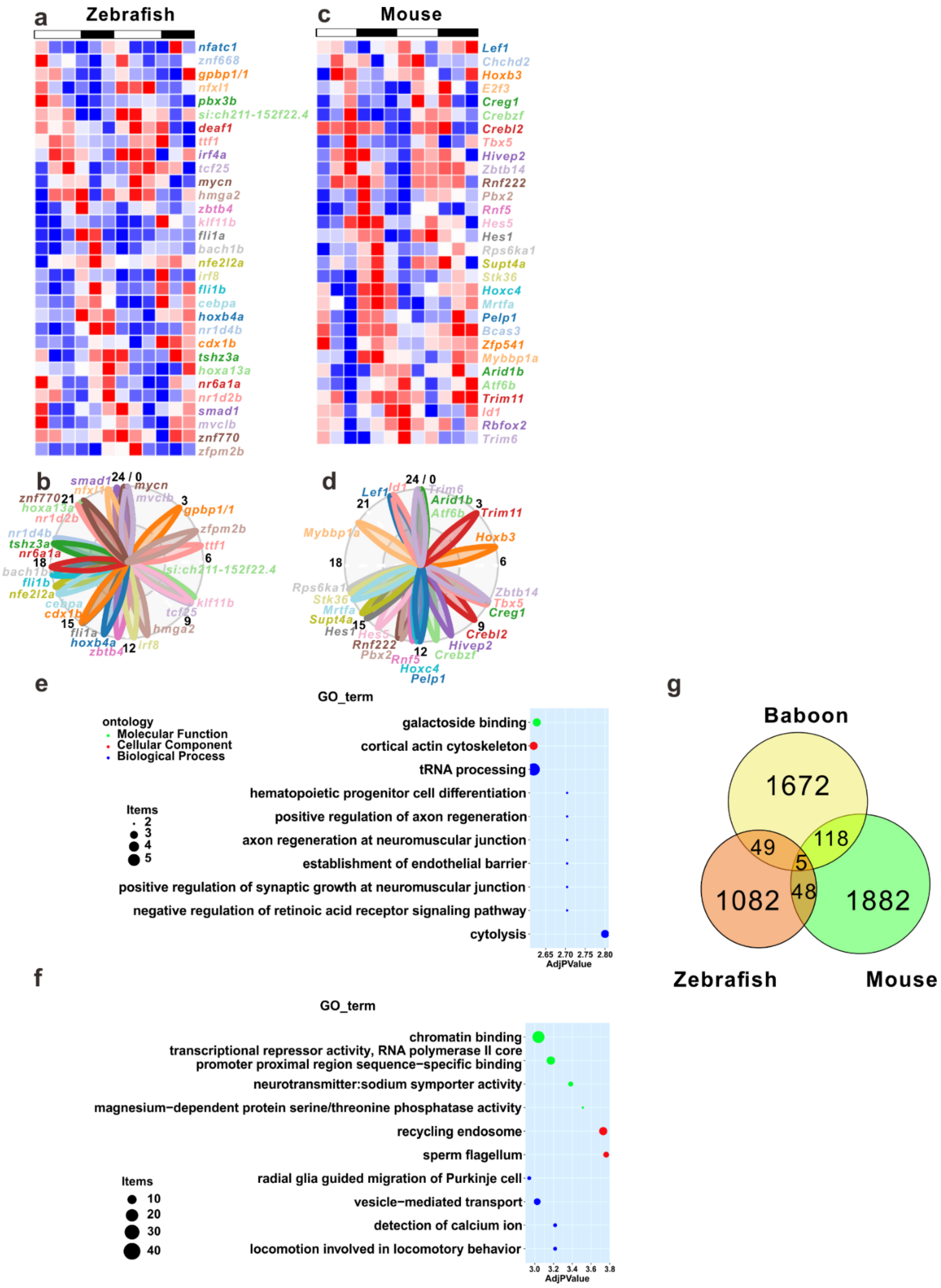

Supplementary Fig. S1 Rhythmic testicular transcriptome in zebrafish and mice.

(**a-d**) Heatmaps and phases of the rhythmically expressed transcriptional factor genes, as computed and

displayed by BioDare2. The gene names in zebrafish (**a**) and mouse (**c**) and their phases (**b**, **d**) are color-coded and annotated. (**e**-**f**) Top-10 enriched GO terms of the rhythmically expressed genes in the zebrafish (**e**) and mouse (**f**) testes. The number of genes in GO terms BP (Biological Process), CC (Cellular Component), and MF (Molecular Function) are indicated by blue, red, and green circles, respectively. (**g**) Venn diagram of the rhythmically expressed testicular genes of zebrafish, mice, and baboons.

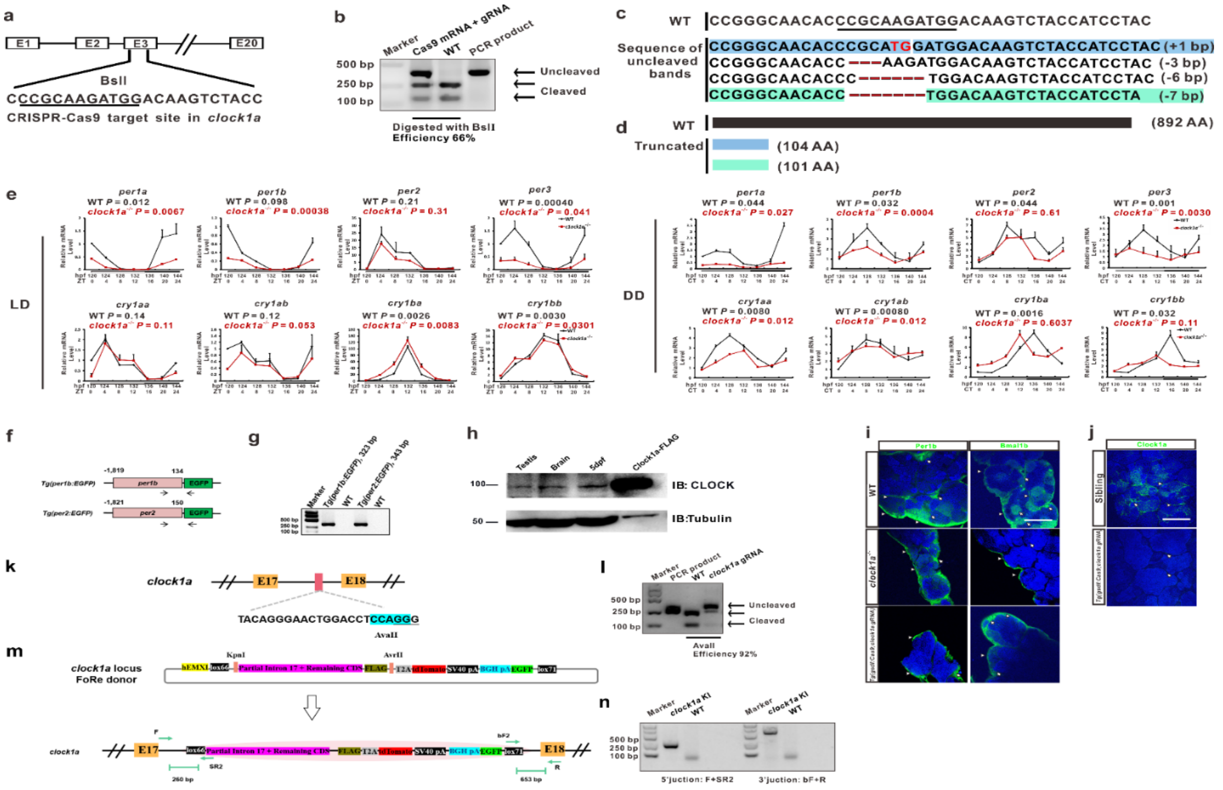

Supplementary Fig. S2 Generation of *clock1a$^{-/-}$* mutant zebrafish, *Tg(per1b:EGFP)* and *Tg(per2:EGFP)* transgenic zebrafish, and *clock1a* knock-in zebrafish.

(**a**) The gRNA target site (underlined) in exon 3 of *clock1a* with a BslI restriction site. (**b**) Determination of the mutagenesis efficiency with PCR and BslI digestion, following microinjection of *clock1a* gRNA and *Cas9* mRNA into one-cell embryos. (**c**) Four heritable zebrafish *clock1a* mutant lines after screening F_1 with PCR amplification of the targeted fragment and sequencing analysis. (**d**) Homozygous *clock1a$^{-/-}$* mutant zebrafish lines with the 1-bp indel or 7-bp deletion, both resulting in truncated proteins. The 7-bp deletion mutant with only 101 amino acids was used for the experiments. (**e**) Disrupted expression of *per1a*, *per1b*, *per2*, *per3*, *cry1aa*, *cry1ab*, *cry1ba*, and *cry1bb* in the *clock1a$^{-/-}$* under LD and DD conditions, shown by qRT-PCR analyses (n = 3 X 3). Expression levels were normalized to *β-actin* and analyzed by JTK_Cycle. All error bars are ± S D. (**f**) Generation of the *Tg(per1b:EGFP)* and *Tg(per2:EGFP)* transgenic zebrafish lines. Schematic diagrams of the DNA constructs wherein EGFP is driven by the *per1b* or *per2* promoter. (**g**) Identification of transgenic *Tg(per1b:EGFP)* and *Tg(per2:EGFP)* zebrafish lines by PCR. (**h**) Western blotting assays using mouse CLOCK antibody and proteins extracted from zebrafish testes, brains, 5-day larvae, and HEK293 cells expressing FLAG-tagged Clock1a. (**i**) Expressions of Bmal1b and Per1b in WT, *clock1a$^{-/-}$* and Sertoli cell *clock1a* mutant testes, as shown by IHC images with Bmal1b and Per1b antibodies (n = 3 X 3). All nuclei were counterstained with Hoechst 33342. Scale bar, 100 μm. (**j**) Down-regulation of Clock1a in Sertoli cells of Sertoli cell *Tg(gsdf:Cas9;CG;u6a:clock1a gRNA;LC) clock1a* mutant testis, as shown by IHC images with the mouse CLOCK antibody (n = 3 X 2). Arrowheads indicate Sertoli cells and arrows spermatogonia. All nuclei were counterstained with Hoechst 33342. Scale bar, 100 μm. (**k**) The gRNA target site (underlined) in Intron 17 of *clock1a* with a AvaII restriction site (cyan). (**l**) Determination of the mutagenesis efficiency with PCR and AvaII digestion, following microinjection of *clock1a* gRNA and *Cas9* mRNA into one-cell embryos. (**m**) Schematic diagram of the KI strategy at the *clock1a* locus based on

the dual-function FoRe donor consisting of two parts. The Forward part is for maintaining the function of the *clock1a* gene, and the Reverse part is for disruption of the *clock1a*. 5' terminal was detected by PCR with F and SR2 primers (260 bp), and 3' terminal was detected by PCR with bF and R primers (653 bp). (**n**) Identification of *clock1a* knock-in zebrafish by junction PCRs.

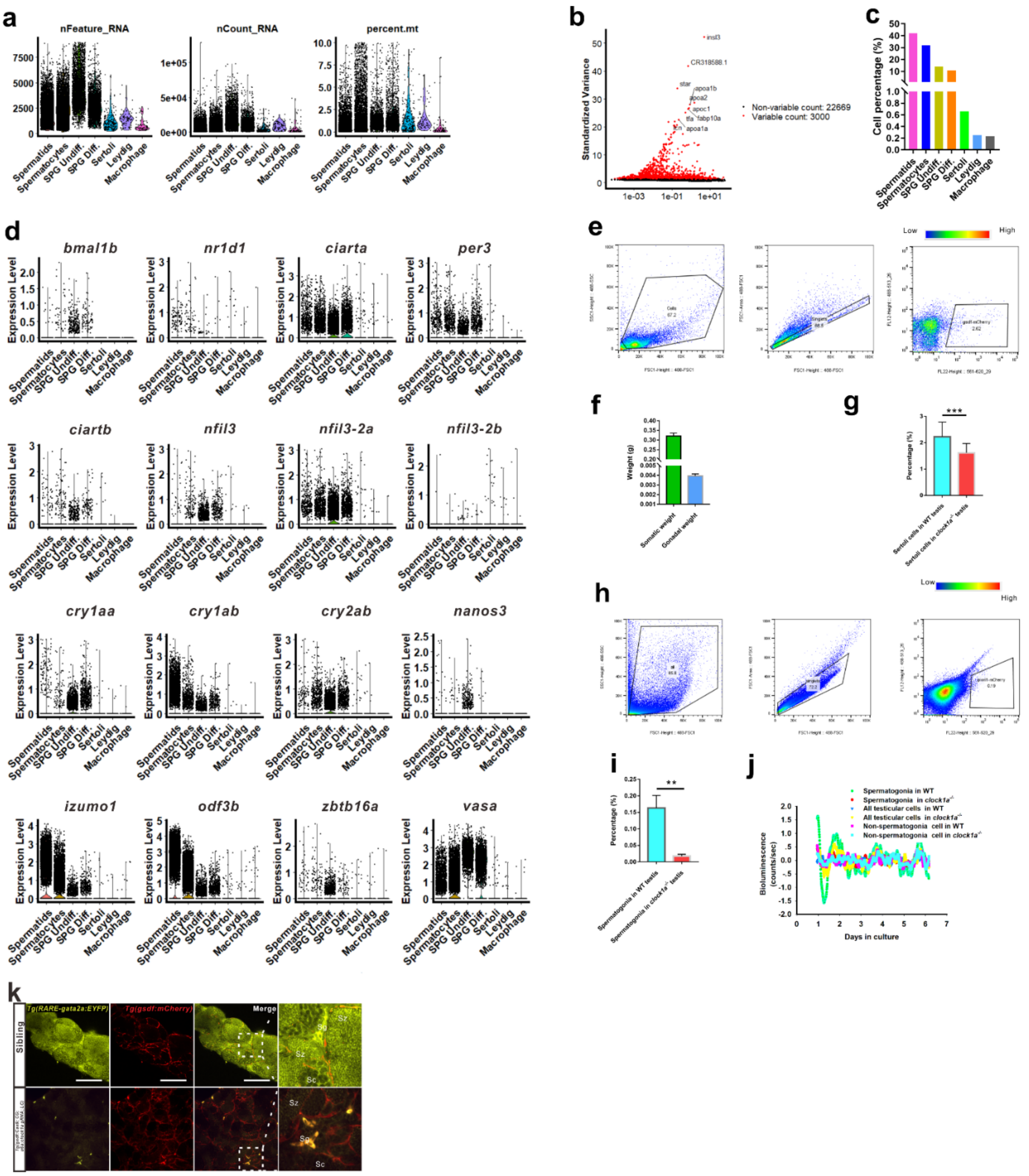

Supplementary Fig. S3 Single-cell RNA-seq analysis of the zebrafish testis and FACS analysis of mCherry-positive Sertoli cells and mCherry-positive spermatogonia.

(**a**) Violin plots show the distribution of the number of genes expressed per cell in different zebrafish testicular cell clusters (left), the total amount of RNA expression per cell in different zebrafish testis clusters with a main population lower than 9,000 counts per cell (middle), and the percentage of the mitochondrial gene tested per cell in zebrafish testicular cell clusters, as shown that most cells were found to have 2% mt-genes or less (right). (**b**) 3,000 highly variable genes in the zebrafish testis were normalized by the variance-stabilizing transformation (vst) method based on variance and mean values using local polynomial regression. (**c**) The percentages of cell clusters in the zebrafish testis determined by scRNA-seq analysis. (**d**) Violin plots show the expression level of circadian clock genes and reproduction-related genes in

zebrafish testicular cell clusters. (**e**) Selection of mCherry-positive Sertoli cells by FACS. Shown are all cells, singlets (middle), and how mCherry-positive cells were isolated (right). (**f**) Statistics of somatic weight and gonadal weight in 3-month-old male zebrafish. (**g**) Estimated Sertoli cells in the testes of 3-month-old WT and *clock1a*$^{-/-}$ male zebrafish. (**h**) Selection of mCherry-positive spermatogonia by FACS. Shown are all cells (left), singlets (middle), and how mCherry-positive cells were isolated (right). (**i**) Estimated spermatogonia in the testes of 3-month-old WT and *clock1a*$^{-/-}$ male zebrafish. (**j**) Bioluminescence analysis of cells isolated from disassociated testes of *Tg(per3:luc;piwil1:mCherry)* and *Tg(per3:luc;piwil1:mCherry);clock1a*$^{-/-}$ zebrafish line by FACS. Rhythmicity of the bioluminescence signal driven by *per3* was not observed in spermatogonia ($n = 3$). ** indicates $P < 0.01$, and *** $P < 0.001$. (**k**) Reduced RA in Sertoli cell-specific *clock1a* mutant testis, as shown by representative confocal images of the testes from *Tg(RARE-gata2a:NLS-EYFP;gsdf:mCherry;gsdf:Cas9;CG;u6a:clock1a gRNA;LC)* and sibling *Tg(RARE-gata2a:NLS-EYFP;gsdf:mCherry)* male zebrafish ($n = 3$ X 3). The dotted square of the merged image is zoomed in. Sg, spermatogonia; Sc, spermatocyte; Sz, spermatozoon. Scale bar, 100 μm.

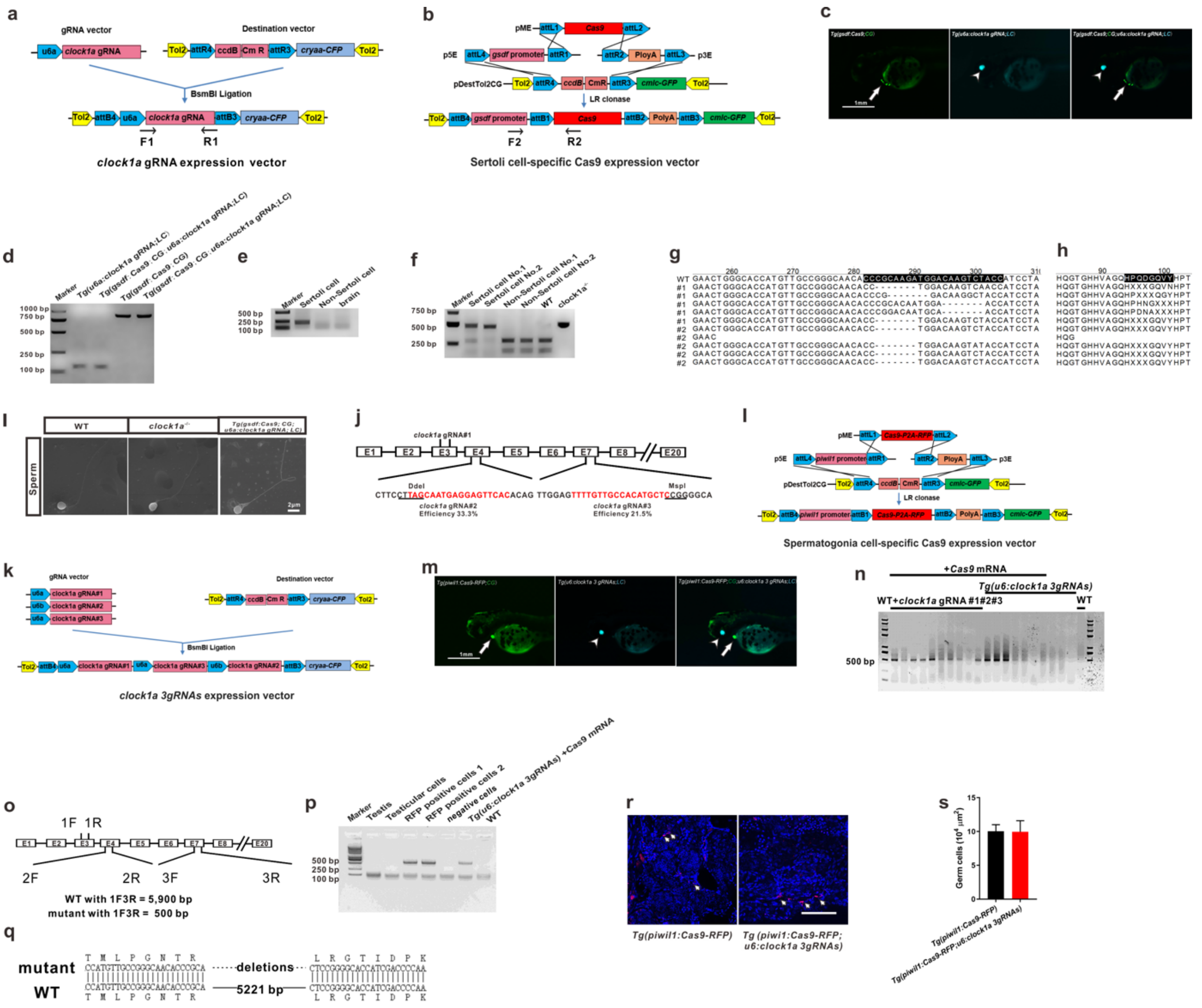

Supplementary Fig. S4 Generation of Sertoli cell-specific *clock1a* mutant and spermatogonia-specific *clock1a* mutant zebrafish.

(**a**) Schematic diagram of the *clock1a* gRNA-expressing vector with LR reaction. (**b**) Schematic diagram of the *gsdf* promoter-driven *Cas9*-expressing vector with LR reaction. (**c**) Identification of heritable *Tg-u6a:clock1a gRNA;LC)* zebrafish by *cryaa*-driven CFP expression in the lens (arrowheads) and *Tg(gsdf:Cas9;CG)* zebrafish by *cmlc*-driven GFP expression in the heart (arrows). The CFP- and GFP-positive larvae were further confirmed by PCR and sequencing analyses. Scale bar, 1 mm. (**d**) Genotyping *Tg(u6a:clock1a gRNA;LC)* and *Tg(gsdf:Cas9;CG)* zebrafish lines with PCR. (**e**) *Cas9* mRNA detection only in Sertoli cells but not in non-Sertoli cells in the testis or the brain of Sertoli cell *clock1a* mutant *Tg(gsdf:Cas9;CG;u6a:clock1a gRNA;LC)* zebrafish by qRT-PCR. (**f**) Determination of the mutation in Sertoli cell-specific *clock1a* mutant zebrafish with PCR and Bsl1 digestion. DNAs were isolated from FACS-selected Sertoli cells and non-Sertoli cells of Sertoli cell-specific *clock1a* mutant, WT, and the $clock1a^{-/-}$ testes, respectively. Two transgenic zebrafish lines were identified. (**g**) DNA sequencing of the *clock1a* gRNA targeted fragment. The targeted fragment was PCR amplified from FACS-selected Sertoli cells of the Sertoli cell-specific *clock1a* mutant zebrafish and sequenced (n = 2 X 5). The targeted wild-type DNA sequence is on the top (black). (**h**) Mutated peptides in the targeted fragments corresponding clones in **g**. The targeted wild-type amino acid sequence is on the top (black). (**i**) SEM (scanning electron microscope) images of the sperms of WT control, $clock1a^{-/-}$, and Sertoli cell-specific *clock1a* mutant zebrafish lines (n = 13-20). Scale bar, 2 μm. (**j**) Schematic diagram of three gRNA sites and their efficiencies identified for the *clock1a 3gRNAs* expressing vector. The efficiency of gRNA#1 is shown in Supplementary Fig. S2b. (**k**) Schematic diagram of the *clock1a 3gRNAs* expressing vector with LR reaction. (**l**) Schematic diagram of the *piwil1* promoter-driven *Cas9*-expressing vector with the RFP tag by LR reaction. (**m**) Identification of heritable *Tg(u6:clock1a 3gRNAs;LC)* zebrafish by *cryaa*-driven CFP expression in the lens (arrowheads)

and *Tg(piwil1:Cas9-RFP;CG)* zebrafish by *cmlc*-driven GFP expression in the heart (arrows). The CFP- and GFP-positive larvae were further confirmed by PCR and sequencing analysis. Scale bar, 1 mm. (**n**) Genotyping embryos after microinjecting *Cas9* mRNA to WT embryos with three *clock1a* gRNAs or to *Tg(u6:clock1a 3gRNAs;LC)* transgenic embryos with primers #1F and #3R. Note that the 500-bp fragment was PCR amplified with DNAs from potential *clock1a* mutant embryos. (**o**) Schematic diagram of the primers flanking three gRNA target sites. Note that the PCR fragment using wild-type zebrafish DNAs is predicted to be 5,900 bp, which is too big to be amplified with the same PCR profile. (**p**) Determination of the mutation in spermatogonia-specific *clock1a* mutant *Tg(piwil1:Cas9-RFP;CG;u6:clock1a 3gRNAs;LC)* zebrafish with PCR primers #1F and #3R. (**q**) Spermatogonia-specific *clock1a* mutant has a 5,221-bp deletion, including partial exon 3, exon 4, exon 5, exon 6, and partial exon 7, as confirmed by DNA sequencing analysis. (**r**) Fluorescent images of RFP signals in *Tg(piwil1:Cas9-RFP)* and spermatogonia-specific *clock1a* mutant testes (*n* = 5). (**s**) Quantification of fluorescent images **r** (*n* = 3).

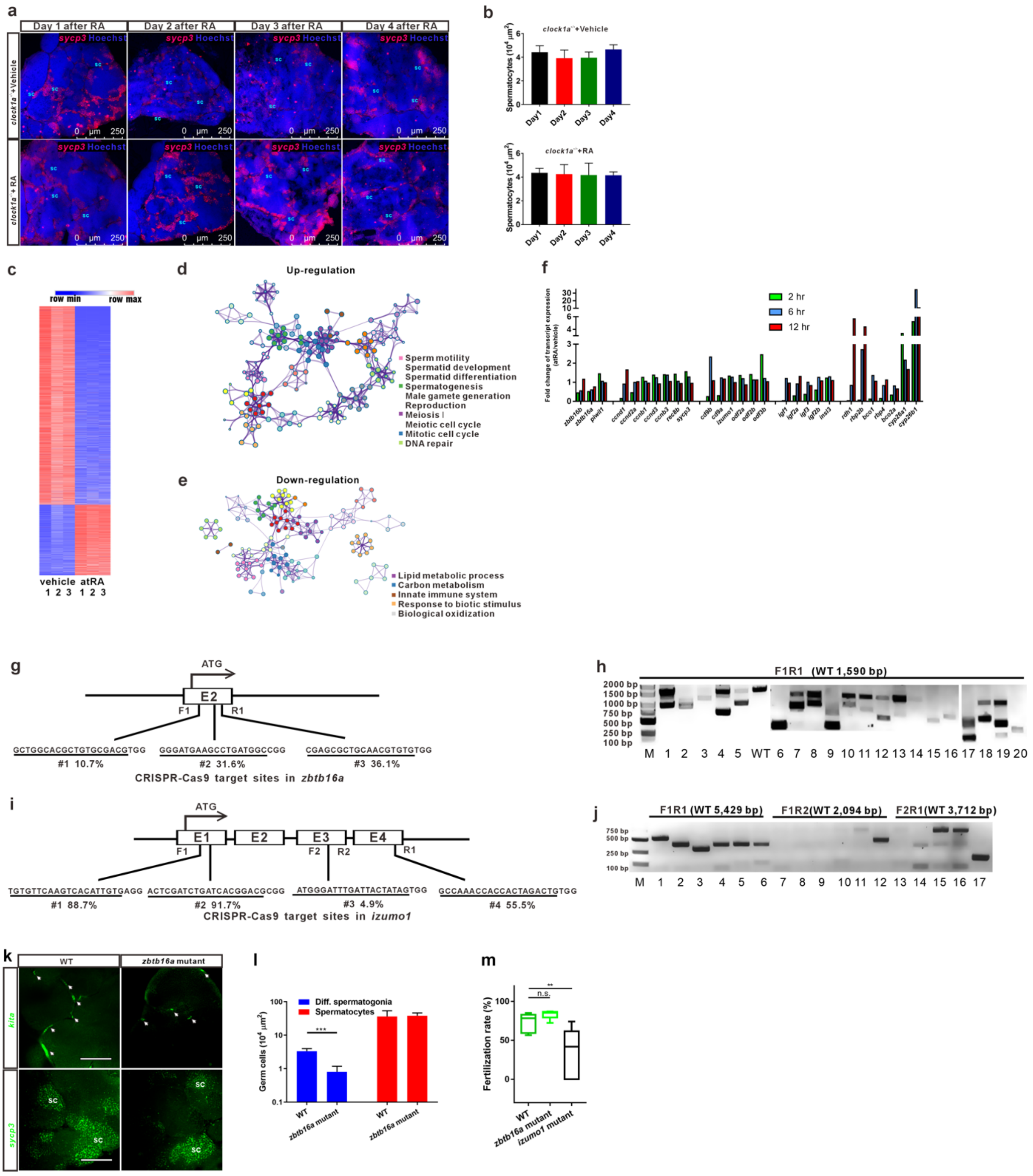

Supplementary Fig. S5 Effect of atRA treatment on spermatocytes and generation and characterization of *zbtb16a* mutant and *izumo1* mutant zebrafish.

(**a**) FISH images of the wild-type and *clock1a*$^{-/-}$ testicular sections with the *sycp3* probe on Days 1-4 following the atRA or vehicle treatment *i.p.* at ZT12 (n = 3 X 5). The signals were superimposed with the Hoechst33423 nuclear counterstain. Scale bar, 250μm. (**b**) Quantification of the signals of the *sycp3*-positive cells in **A** (n = 4). (**c**) Heatmap of the differentially expressed genes revealed by the transcriptome analysis of the *clock1a*$^{-/-}$ testis 2 hours after the atRA or vehicle treatment (P < 0.05, n = 3). (**d-e**) Functional annotation of genes in the *clock1a*$^{-/-}$ mutant testis significantly altered by the atRA treatment, representatives from up-regulated 1,813 genes (**d**) and down-regulated 5,034 genes (**e**) using the ClueGO plugin from Metascape. Nodes represent the individual GO terms, edges the correlation between two nodes. The nodes are grouped into different colors for the upper-level GO terms. (**f**) Ratios of expression levels of genes

involved in testicular functions in the *clock1a*$^{-/-}$ testes treated with atRA to those treated with the vehicle. A value higher than one indicates that the gene expression is higher in the atRA group and *vice versa*. Transcriptome analysis of the *clock1a*$^{-/-}$ testes collected two, six, and 12 hours after the atRA or vehicle treatment was performed. (**g**, **i**) Schematic diagrams of the three CRISPR-Cas9 target sites (underlined) in exon 2 of the *zbtb16a* locus (**g**), and the four CRISPR-Cas9 target sites (underlined) in exon 1, exon 3, and exon 4 of the *izumo1* locus (**i**). The *zbtb16a* gRNA and *izumo1* gRNA efficiencies were estimated by the TIDE analysis of the PCR products, respectively. (**h**, **j**) Genotyping the *zbtb16* or *izumo1* mutant zebrafish by PCR. Compared with the 1,590-bp band in wild types, those with smaller bands indicate *zbtb16a* deletion mutants (**h**). Compared with the projected bands of 5,429 bp (F1R1), 2,094 bp (F1R2), and 3,712 bp (F2R1) (not shown) in wild types, those with smaller bands indicate *izumo1* deletion mutants (**j**). Fin-clipped DNAs from individual adult zebrafish were used. (**k**) FISH images with WT control and the *zbtb16a* mutant testes, a *kita* antisense probe, and a *sycp3* antisense probe (n = 3 X 5). Arrow, spermatogonia; Sc, spermatocyte. Scale bar, 100 µm. (**l**) Quantification analysis of FISH images of the *zbtb16a* mutant testes in **k** (n = 5-18). (**m**) Fertilization rates of pairwise crosses of wild-type females and the *zbtb16a* mutant males and *izumo1* mutant males (n = 5-11). ** indicates $P < 0.01$, and *** $P < 0.001$.

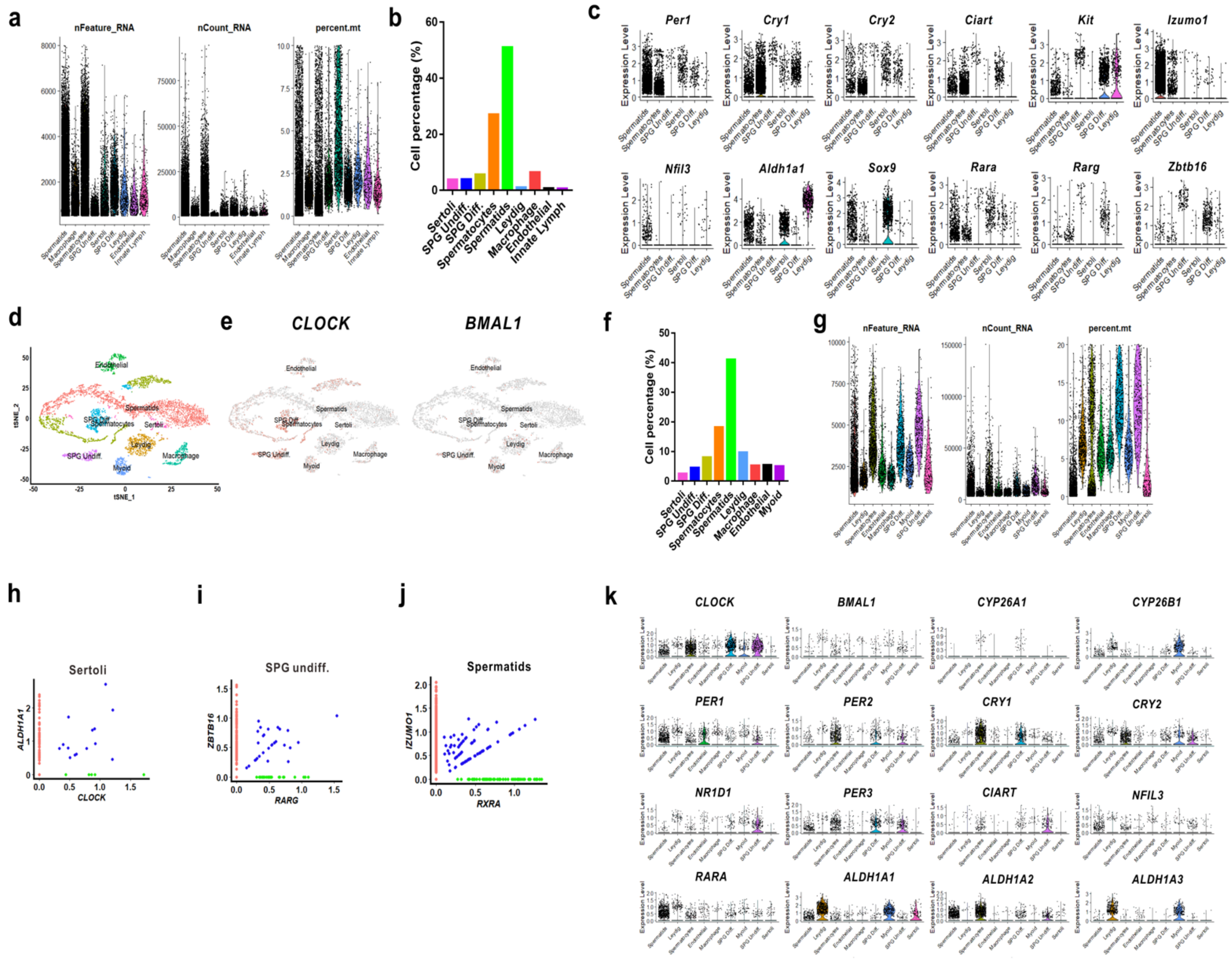

Supplementary Fig. S6 Single-cell RNA-seq analysis of the testes of mice and humans.
(**a**) Violin plots show the distribution of the number of genes expressed per cell in different mouse testis clusters (left), the total amount of RNA expressions per cell in different mouse testis clusters with the main population lower than 9,000 counts per cell (middle), and the percentage of the mitochondrial gene tested per cell in different mouse testis clusters (right). (**b**) The percentages of cell clusters in mouse testis, determined by scRNA-seq analysis. (**c**) Violin plots show the expression levels of circadian clock genes and reproduction-related genes in distinct mouse testicular cell clusters. (**d**) scRNA-seq analysis identifies the nine human testicular clusters. (**e**) The expression (red dots) of circadian clock genes *CLOCK* and *BMAL1* in the human testicular cell clusters. (**f**) The percentages of cell clusters in human testis, determined by the scRNA-analysis. (**g**) Violin plots show the distribution of the number of genes expressed per cell in different human testicular cell clusters (left), the total amount of RNA expressions per cell in different human testis clusters with main population lower than 15,000 counts per cell (middle), and the percentage of the mitochondrial gene tested per cell in different human testicular cell clusters (most cells were found to have15% mt-genes or less) (right). (**h-j**) Single-cell co-expressions (blue dots) of *ALDH1A1* (red dots) with *CLOCK* (green dots) in Sertoli cells (**h**), *ZBTB16* (red dots) with *RARG* (green dots) in SPG undiff. (**i**), and *IZUMO1* (red dots) with *RXRA* (green dots) in spermatids (**j**). (**k**) Violin plots show the expression level of the circadian clock genes and reproduction-related genes in distinct human testicular cell clusters. Scattering dots represented the cells clustering in testicular cell clusters.

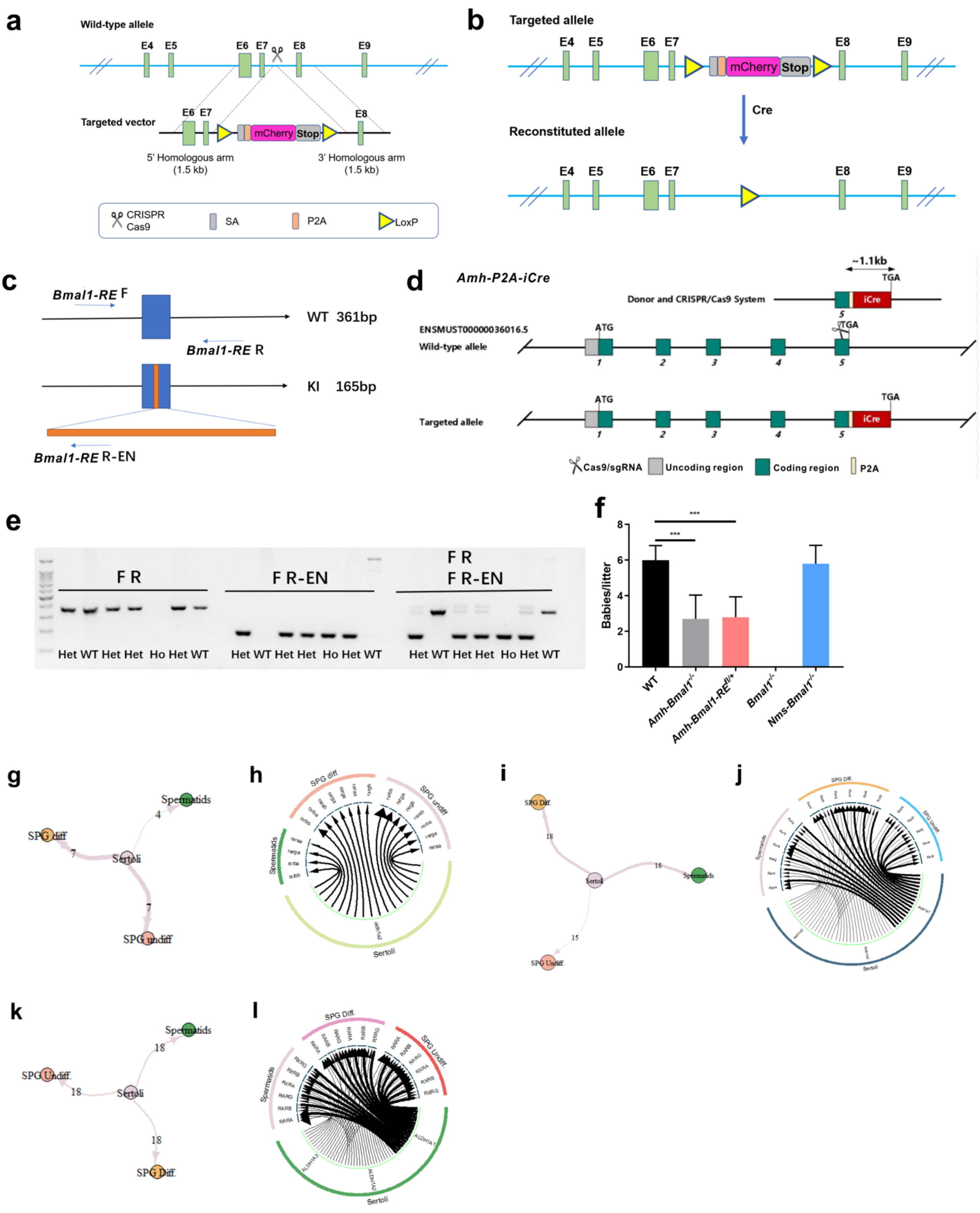

Supplementary Fig. S7 Generation of *Bmal1*-RE and *Amh-P2A-iCre* knock-in mouse lines and network and Circos analyses of cell-cell communications in the testes of zebrafish, mice, and humans. (**a**) Schematic diagram of the design of the *Bmal1*-RE mouse. (**b**) Reconstituted allele after Cre-mediated recombination. (**c**) The location of the primers used for identifying *Bmal1*-RE mice. (**d**) The design and generation of the *Amh-P2A-iCre* knock-in mouse line. (**e**) PCR identification of *Bmal1*-RE mice. (**f**)

Average clutch size per litter in WT, *Amh-Bmal1*$^{-/-}$, and *Amh-Bmal1-RE*$^{fl/fl}$ mice, *Bmal1*$^{-/-}$, and *Nms-Bmal1*$^{-/-}$ (n = 10). *** indicates P < 0.001. (**g, i, k**) Network plot of cell-cell communications from Sertoli cells to spermatogonia or spermatids with respect to RA signaling in zebrafish (**g**), mouse (**i**), and human (**k**). The number of ligand-receptor interactions detected between each cell type is shown. (**h**, **j**, **l**) Circos plot of cell-cell communications among Sertoli cells and spermatogonia or spermatids with respect to RA signaling in zebrafish (**h**), mouse (**j**), and human (**l**). Shown are the top 54 highly expressed ligand-receptor interactions. The arrow indicates the direction of the ligand-receptor interaction, in which the arrow points to the receptor, and the node refers to the ligand. The thickness of the line indicates the relative expression levels from high to low.

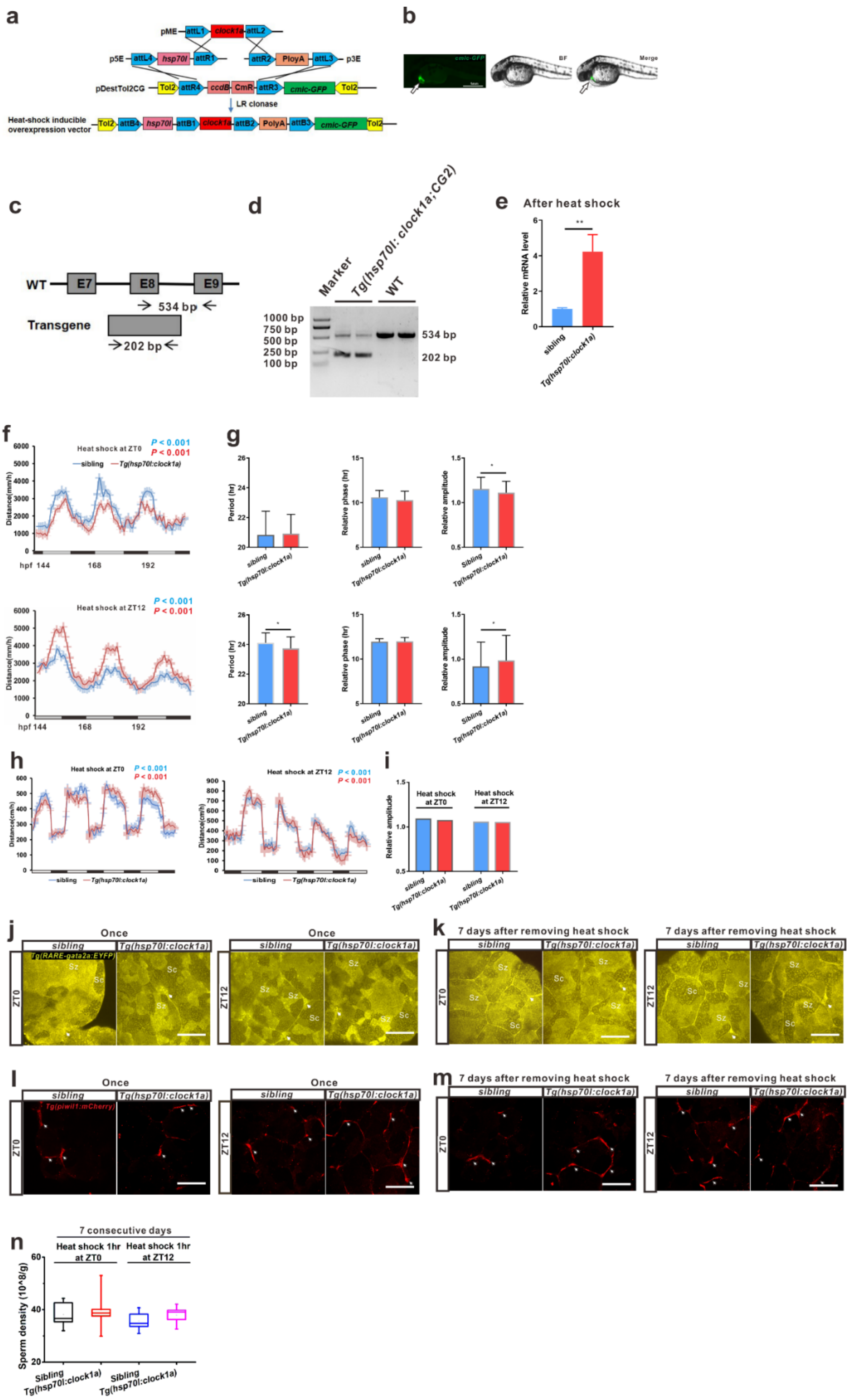

Supplementary Fig. S8 Generation and characterization of *Tg(hsp70l:clock1a;CG)* zebrafish.
(**a**) Schematic diagram of the *clock1a*-overexpressing vector with LR reaction. (**b**) Identification of heritable *Tg(hsp70l:clock1a;CG)* zebrafish by *cmlc*-driven GFP expression in the heart (arrow). Scale bar, 1 mm. (**c-e**) Identification of *Tg(hsp70l:clock1a;CG)* zebrafish with PCR with primers indicated (**c**). The amplified 202-bp fragment indicates the transgene (**d**). Up-regulation of *clock1a* mRNA level in

Tg(hsp70l:clock1a;CG) larvae after heat shock for 1 hr at ZT0 (**e**). (**f**) Disrupted locomotor rhythms of *Tg(hsp70l:clock1a;CG2)* larvae under DD condition following heat shock at 37 °C for one hour starting at ZT0 or ZT12 (n = 24). (**g**) Statistics of the periods, phases, and amplitudes of locomotor activities of *Tg(hsp70l:clock1a;CG2)* larvae following heat shock at 37 °C for one hour starting at ZT0 or ZT12 (n = 4 X 24). (**h-i**) Locomotor rhythms of *Tg(hsp70l:clock1a;CG)* larvae and siblings following heat shock at 37 °C for one hour starting at ZT0 (left) and ZT12 (right) (n = 3 X 24) (**h**) and the amplitudes (**i**) under LD condition. * indicates $P < 0.05$, and ** $P < 0.01$. (**j-k**) Confocal images of the testes from *Tg(RARE-gata2a:NLS-EYFP; hsp70l:clock1a;CG2)* and sibling male zebrafish heat shock only once at ZT0 or ZT12 (**j**), and seven days after removing the heat shock following heat shock at ZT0 or ZT12 (**k**) for seven consecutive days (n = 3 X 9). (**l-m**) Confocal images of *Tg(hsp70l:clock1a;CG2;piwil1:mCherry)* and sibling male testes after heat shock at 37 °C for one hour at ZT0 or ZT12 (**l**), and seven days after removing heat shock following heat shock at ZT0 or ZT12 (**m**) for seven consecutive days (n = 3 X 10). Arrows indicate spermatogonia. SC, spermatocytes. Scale bar, 100 μm. (**n**) Sperm densities of *Tg(hsp70l:clock1a;CG2)* males or sibling males following heat shock at 37 °C for one hour starting at ZT0 or ZT12 for seven consecutive days (n = 3 X 2).

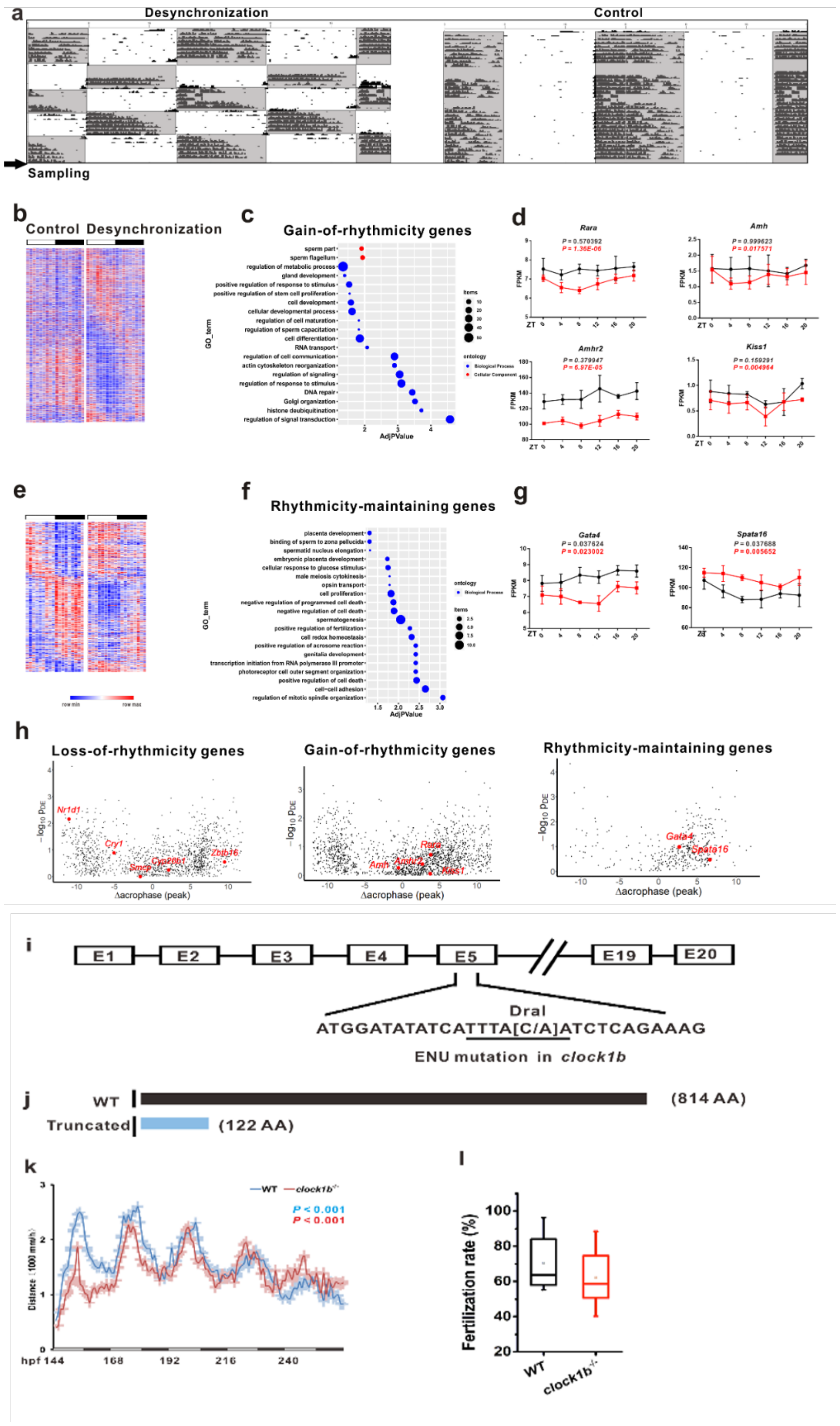

Supplementary Fig. S9 Time-series RNA-seq analysis of the testes of desynchronized and control mice and generation and characterization of *clock1b*$^{-/-}$ mutant zebrafish.

(**a**) The wheel-running plotting of mouse behavior under the desynchronized and light-dark control conditions. (**b**) Heatmaps of gain-of-rhythmicity genes in the control and desynchronization groups. (**c**) Top-

20 enriched GO BP (Biological Process) terms of gain-of-rhythmicity genes in the desynchronization group. (**d**) Expression patterns of *Rara*, *Amh*, *Amhr2,* and *Kiss1* in the desynchronized and control groups. (**e**) Heatmaps of rhythmicity-maintaining genes in the desynchronization and control groups. (**f**) Top-20 enriched GO BP terms of rhythmicity-maintaining genes in the desynchronization group. (**g**) Expression patterns of representative genes *Gata4* and *Spata16* in the desynchronized and control groups. In **c** and **f**, the numbers of genes were indicated by the diameter of the circles. In **d** and **g**, the corresponding rhythmicity *P* values of the MetaCycle analysis are shown above the plots. **(h)** Limorhyde analysis of circadian transcriptome data of mouse testis from control and desynchronization group. Scatterplot of -log 10 (P_{DE}) vs. Δacrophase. P_{DE} corresponds to a rhythmic gene's *P*-value of differential rhythmicity. Δacrophase corresponds to the change in rhythm peak time, where a positive value indicates an advanced phase in the desynchronization group. Each point represents a gene and the representative rhythmic genes in each group. *Cry1*, *Nr1d1*, *Zbtb16*, *Smcp*, and *Cyp26b1* are highlighted in loss-of-rhythmicity genes (left), *Amh*, *Amhr2*, *Rara* and *Kiss1* are marked in gain-of-rhythmicity genes (middle), and *Gata4* and *Spata16* are labeled in rhythmicity-maintaining genes (right). The rhythmic *P* value differences and acrophase changes were calculated by Limorhyde. (**i**) Generation of the *clock1b* mutant by ENU. An ethylnitrosourea (ENU)-induced *clock1b* (ENSDARG00000003631) mutant line (sa386) was recovered from the Zebrafish Mutation Project (http://www.sanger.ac.uk/resources/zebrafish/zmp/) and available from the Zebrafish International Resource Center (ZIRC) (https://zebrafish.org/home/guide.php). A nonsense mutation of C to A occurred in the fifth exon of *clock1b* resulting in a premature stop codon (TAC→TAA). (**j**) The premature stop codon (TAC→TAA) produced a truncated peptide with only 122 amino acids. (**k**) Locomotor analysis of 5 to 9-dpf *clock1b*$^{-/-}$mutant (red) and wild-type control (WT) (black) larvae under DD condition (n = 1 X 24).
(**l**) Fertilization rates of pairwise crosses wild-type females and *clock1b*$^{-/-}$ mutant or wild-type (WT) control males (n = 8). * indicates $P < 0.05$.

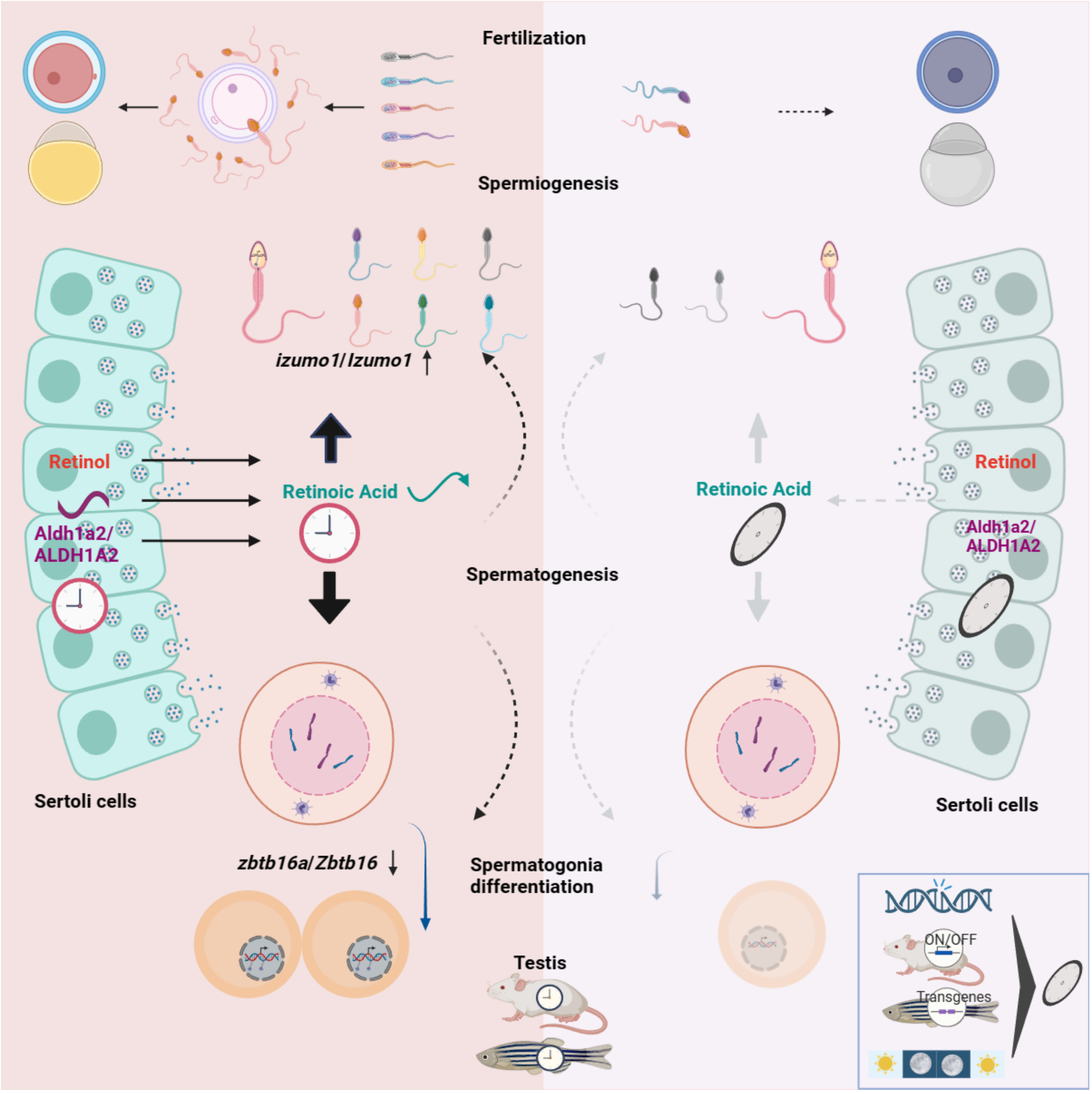

Supplementary Fig. S10 model for the Sertoli cell circadian clock. A putative model for the Sertoli cell clock synchronizes spermatogonial differentiation and promotes fertilization via retinoic acid signaling. The circadian clock in somatic Sertoli cells regulates RA production and RA signaling. RA synthesized in Sertoli cells is diffused to spermatogonia or spermatozoa and exerts its paracrine roles, respectively, *i.e.*, the clock-controlled RA, on the one hand, is to synchronize spermatogonia differentiation via down-regulating transcription suppressor *zbtb16a* by RA; on the other hand, to promote fertilization via up-regulating *izumol1* by RA.

Supplementary Tables S1 to S5

Supplementary Table S1. RNA-seq and rhythmicity analysis of zebrafish testis in two consecutive days with a 4-hour interval.

Supplementary Table S2. RNA-seq and rhythmicity analysis of mouse testis in two consecutive days with a 4-hour interval.

Supplementary Table S3. scRNA-seq analysis and marker genes of testicular clusters in zebrafish.

Supplementary Table S4. RNA-seq analysis in *clock1a*$^{-/-}$ testis after atRA or vehicle treatment.

Supplementary Table S5. RNA-seq and rhythmicity analysis of mouse testis in control and desynchronization groups in triplicate from one consecutive day with a 4-hour interval.

Supplementary Table S6. List of PCR primers and gRNA sites

Primer	**Sequence 5'-3'**	**Application**
clock1a F	AGCAGGGACAGAACCAGG	qRT-PCR
clock1a R	GTGTTGCGGTTGTGAATG	
b-actin F	ACGAACGACCAACCTAAACTCT	qRT-PCR
b-actin R	TTAGACAACTACCTCCCTTTGC	
aldh1a2 F	CCGACAAGGCTGATGTGGAT	qRT-PCR
aldh1a2 R	CAGTAGCTTCCCCCGTTCAG	
cry1aa F	GACGCACAGCAGATAACAGGACG	qRT-PCR
cry1aa R	CAAAATGTAACAACTCGGGAAAAGG	
cry1ab F	TCGCCAAGTGCATAATTGGA	qRT-PCR
cry1ab R	GTGTGTCTCCCGAGGAAGGA	
cry1ba F	TCTACCAACAACTGTCCCGCTAC	qRT-PCR
cry1ba R	GCCATCCCATTTCCATTCCC	
cry1bb F	TGGGCGGGACTGGAAAAA	qRT-PCR
cry1bb R	TGGTGGGTGGGGCTTAGGTA	
per1a F	GGAAAAGGCTCAGCCACAGA	qRT-PCR
per1a R	TGAACTTCCGCTCAAAAGAC	
per1b F	AGGAAGGCTGACAGATGATGAATG	qRT-PCR
per1b R	CCAGAGTGGGCTAAAGCGAAGTA	
per2 F	ACGAGGACAAGCAGAGGAACG	qRT-PCR
per2 R	GCACTGGCTGGTGATGGAGA	
per3 F	GTTCTGGCGGAGTAGTGGAG	qRT-PCR
per3 R	TGACGACGTTTTACTGGTGC	
aldh1a2ChIP F	TTTAACGCATCACCAGAGCC	

aldh1a2ChIP R	GGTGCGCACACACACAAAC	Enhancer cloning and ChIP
rarga ChIP F	ATATTTATCAGCTTTGATTC	Enhancer cloning and ChIP
rarga ChIP R	CGATCGAGTCTGAAACCACC	
per1b promoter F	AAGCCTGAGGACACATTGCT	Promoter cloning
per1b promoter R	TTCTGTTGAACGACAAGAC	
per2 promoter F	TTCCCAGTGCTTAGTGGCAG	Promoter cloning
per2 promoter R	TAACACCGCTGCCCTGGATC	
per1b promoter identify F	CAACAGGCGCTCTGCAACGT	Genome PCR
per2 promoter identify F	TACCCAATAGGAGCGCTCGG	Genome PCR
EGFP identify R	CGGACACGCTGAACTTGTG	Genome PCR
clock1a probe F	AGTCTCAGTTGAACACCTCCAGC	Probe
clock1a probe R	GCAGTGTGTGGGTCGACCTC	
kita probe F	CGGCTTGACTCTGGACACTG	Probe
kita probe R	CACCATCACTGTCCTTTCTT	
izumo1 promoter F	TGCTGATTGGCTCACAGATAGAACC	Promoter cloning
izumo1 promoter R	CCCTATCGACGGATAGGGTAGGTAT	
zbtb16a promoter F	CCTAAATCTAGCACTAAACCCG	Promoter cloning
zbtb16a promoter R	GTGTTGCGCGTTTGGCTTCC	
clock1a cDNA F	GACCTCCAGCATAGACCGGG	cDNA
clock1a cDNA R	ACTGAGGCGGAGGGTTGGAG	
gsdf promoter F	ACCCAGGCCACCATTACAGAACT	Promoter cloning
gsdf promoter R	ATGTCTGTGGATTCAGGAGCGT	
clock1a gRNA site	GGTAGACTTGTCCATCTTGC	gRNA synthesis
Cas9-P4	AAAAGCACCGACTCGGTGCC	gRNA synthesis
clock1a-7bp F	TTGCTGGATTCCTCTGAGATTAG	Genome PCR

clock1a-7bp R	GAATTCGCCCACCC TATTTT	
clock1a #2 F	AATTGCTGCACAGT CGGAGT	Genome PCR
clock1a #2 R	CCATCCGTCATAAT GGCCAGA	
clock1a #3 F	GTGTTCCATTTGGG ACGACG	Genome PCR
clock1a #3 R	TCTGGCTTTTCTTTG CAGCG	
gsdf promoter identify F	TTGTATCAGAGAGT GATGAA	Genome PCR
izumo1 probe F	TTGTATCAGAGAGT GATGAA	RNA Probe
izumo1 probe R	TTCAAACTGGAACA GTAGAA	
zbtb16a probe F	TCATCCAGAGGGAG TTCTTC	RNA Probe
zbtb16a probe R	TGATAAATCCGTCC ATTTCC	
Cas9 F	CTGCCGAAGCAACA CGCCTG	RT-PCR
Cas9 R	AGGCGACCTCGTCC ACAATG	
zbtb16a gRNA site #1	GCTGGCACGCTGTG CGACG	gRNA synthesis
zbtb16a gRNA site #2	GGGATGAAGCCTG ATGGC	gRNA synthesis
zbtb16a gRNA site #3	CGAGCGCTGCAACG TGTG	gRNA synthesis
izumo1 gRNA site #1	TGTGTTCAAGTCAC ATTGTG	gRNA synthesis
izumo1 gRNA site #2	ACTCGATCTGATCA CGGACG	gRNA synthesis
izumo1 gRNA site #3	ATGGGATTTGATTA CTATAG	gRNA synthesis
izumo1 gRNA site #4	GCCAAACCACCACT AGACTG	gRNA synthesis
clock1a-E8-F	TGCCTAACTCAACA CGAAAC	Genome PCR
clock1a-E9-R	CTGTGGTCCAAGAA GAGGAA	
clock1b F	TCAGCAATGAGGA GTTTTCGC	Genome PCR
clock1b R	CCACTGGAAGAAA GTTTAGAAGGT	
zbtb16a F	ATTGACCTTATCTT GTTTAC	Genome PCR
zbtb16a R	AATCAACAGTTAAC CATCAT	

izumo1 F1	GATATTTCGTGCCT TGTGATGATG	Genome PCR
izumo1 R1	TGCTCTGAACGAAT AGAAAGCA	
izumo1 F2	ATCCTACAACACTC TATCGT	Genome PCR
izumo1 R2	CACATTTATTAGGA CACAGA	
KI-sR2	GGTACCTTTCCATT ACAAGCGCCGCTC	Identification of Knock-in zebrafish
KI-F	GGAAAGAGGTACA AACCGTGACTC	
KI-bF	CCTGCGTTATCCCC TGATTC	
KI-R	TTGGAACTAACCCC TGTGCAGA	
XM003950- Amh-5TF1	CGTTCTATTCCAGA CTGTCCCTGGA	Genome PCR
XM003678- Adipoq-5TR1	CACTCTGATTCTGG CAATTTCGG	
XM003950- Amh-TF1	CTACCAAGCCAACA ACTGCCAAG	
XM003950- Amh-TR2	GAGTGAGTTAGAGT TCCAGGACCACC	
Bmal1-RE F	GCCTCCATCAGACA GAAGTGA	Genome PCR

Supplementary Table S7. Experimental Models: Organisms and Strains		
Zebrafish: *Tg(per3:luc)*	Kaneko et al., 2005	ZDB-TGCONSTRCT-070117-112
Zebrafish: *Tg(bmal1b:luc)*	Wang et al., 2015	N/A
Zebrafish: *clock1a*$^{-/-}$	This paper	N/A
Zebrafish: *clock1b*$^{-/-}$	This paper	N/A
Zebrafish: *Tg(per2:EGFP)*	This paper	N/A
Zebrafish: *Tg(per1b:EGFP)*	This paper	N/A
Zebrafish: *Tg(nanos3:EGFP)*	A kind gift of Qingshun Zhao	N/A
Zebrafish: *Tg(piwil1:EGFP)*	Leu and Draper, 2010	ZDB-TGCONSTRCT-110126-5
Zebrafish: *Tg(gsdf:mCherry)*	Leerberg et al.,2017	ZDB-TGCONSTRCT-171120-2
Zebrafish: *Tg(piwil1:mCherry)*	A kind gift of Bruce Draper	N/A
Zebrafish: *Tg(hsp70l:clock1a;CG2)*	This paper	N/A
Zebrafish: *zbtb16a mutant*	This paper	N/A
Zebrafish: *izumo1 mutant*	This paper	N/A
Zebrafish: *Tg(RARE-gata2a:NLS-EYFP)*	Perz-Edwards et al., 2001	ZDB-TGCONSTRCT-070117-78
Zebrafish: *Tg(gsdf:Cas9;CG2)*	This paper	N/A
Zebrafish: *Tg(piwil1:Cas9-RFP;CG2)*		
Zebrafish: *Tg(u6a:clock1a gRNA;LC)*	This paper	N/A
Zebrafish: *Tg(gsdf:Cas9;CG2;u6a:clock1a gRNA;LC)*	This paper	N/A
Zebrafish: *Tg(per2:EGFP);clock1a*$^{-/-}$	This paper	N/A
Zebrafish: *Tg(per1b:EGFP);clock1a*$^{-/-}$	This paper	N/A
Zebrafish: *Tg(per3:luc);clock1a*$^{-/-}$	This paper	N/A
Zebrafish: *Tg(RARE-gata2a:NLS-EYFP);clock1a*$^{-/-}$	This paper	N/A
Zebrafish: *Tg(nanos3:EGFP);clock1a*$^{-/-}$	This paper	N/A
Zebrafish: *Tg(piwil1:EGFP);clock1a*$^{-/-}$	This paper	N/A
Zebrafish: *Tg(per2:EGFP;gsdf:mCherry)*	This paper	N/A
Zebrafish: *Tg(per1b:EGFP;gsdf:mCherry)*	This paper	N/A

Zebrafish: *Tg(RARE-gata2a:NLS-EYFP;gsdf:mCherry)*	This paper	N/A
Zebrafish: *Tg(hsp70l:clock1a;CG2;piwil1:mCherry)*	This paper	N/A
Zebrafish: *Tg(RARE-gata2a:NLS-EYFP;hsp70l:clock1a;CG2)*	This paper	N/A
Zebrafish: *Tg(gsdf:mCherry;per3:luc)*	This paper	N/A
Zebrafish: *Tg(piwil1:mCherry;per3:luc)*	This paper	N/A
Zebrafish: *Tg(gsdf:mCherry;per3:luc);clock1a* $^{-/-}$	This paper	N/A
Zebrafish: *Tg(piwil1:mCherry;per3:luc);clock1a* $^{-/-}$	This paper	N/A
Zebrafish: *Tg(per2:EGFP;gsdf:mCherry);clock1a* $^{-/-}$	This paper	N/A
Zebrafish: *Tg(per1b:EGFP;gsdf:mCherry);clock1a* $^{-/-}$	This paper	N/A
Zebrafish: *Tg(RARE-gata2a:NLS-EYFP;gsdf:mCherry);clock1a-/-*	This paper	N/A
Zebrafish: *Tg(per3:luc;gsdf:Cas9;CG2;u6a:clock1a gRNA;LC)*	This paper	N/A
Zebrafish: *Tg(nanos3:EGFP;gsdf:Cas9;CG2;u6a:clock1a gRNA;LC)*	This paper	N/A
Zebrafish: *Tg(piwil1:EGFP;gsdf:Cas9;CG2;u6a:clock1a gRNA;LC)*	This paper	N/A
Zebrafish: *Tg(RARE-gata2a:NLS-EYFP;gsdf:mCherry;gsdf:Cas9;CG2;u6a:clock1a gRNA;LC)*	This paper	N/A
Zebrafish: *Tg(u6a:clock1a 3gRNAs;LC)*	This paper	N/A
Zebrafish: *Tg(gsdf:Cas9-RFP;CG2; u6:clock1a 3gRNAs;LC)*	This paper	N/A
Zebrafish: *Tg(piwil1:Cas9-RFP;CG2; u6:clock1a 3gRNAs;LC)*	This paper	N/A
Zebrafish: *clock1a-KI-tdTomato*	This paper	N/A
Mouse: *Bmal1* $^{-/-}$	Bunger et al., 2000	
Mouse: *Amh-P2A-iCre*	This paper	
Mouse: *Nms-iCre*	Lee et al., 2015	

Mouse: *Bmal*$^{fl/fl}$	Storch et al., 2007	
Mouse: *Bmal1-RE*$^{fl/+}$	This paper	
Mouse: *Rosa26-EGFP*$^{fl/+}$	Mao et al., 2001	
Mouse: *PER2::LUC*	Yoo et al., 2004	
Mouse: *PER2::LUC; Bmal1*$^{-/-}$	This paper	
Mouse: *Amh-P2A-iCre;Bmal1*$^{fl/fl}$	This paper	
Mouse: *PER2::LUC;Amh-P2A-iCre; Bmal1*$^{fl/fl}$	This paper	
Mouse: *Amh-P2A-iCre;Bmal1-RE* $^{fl/fl}$	This paper	
Mouse: *PER2::LUC;Amh-P2A-iCre; Bmal1-RE*$^{fl/+}$	This paper	
Mouse: *Amh-P2A-iCre;Rosa26-EGFP*$^{fl/+}$	This paper	
Mouse: *Nms-iCre; Bmal1*$^{fl/fl}$	This paper	
Mouse: *PER2::LUC; Amh-P2A-iCre; Rosa26-EGFP*$^{fl/+}$	This paper	

Supplementary Movie S1. 3D volume renderings of the testes from *Tg(per1b:EGFP)* (upper left), *Tg(per1b:EGFP);clock1a$^{-/-}$* (upper right), *Tg(per2:EGFP)* (lower left), *Tg(per2:EGFP);clock1a$^{-/-}$* (lower right).

Supplementary Movie S2. Sperms of wild-type (upper), *clock1a$^{-/-}$* (middle), Sertoli cell *clock1a* mutant *Tg(gsdf:Cas9;u6a:clock1a gRNA)* (lower) under a microscope.

Reference:

1. Westerfield M. *The Zebrafish Book. A Guide for the Laboratory Use of Zebrafish (Danio rerio), 5th Edition*: University of Oregon Press, Eugene, 2007.
2. Kaneko M, Cahill GM. Light-dependent development of circadian gene expression in transgenic zebrafish. *PLoS biology*. 2005; **3**(2): e34. doi: 10.1371/journal.pbio.0030034
3. Wang M, Zhong Z, Zhong Y *et al.* The zebrafish period2 protein positively regulates the circadian clock through mediation of retinoic acid receptor (RAR)-related orphan receptor alpha (Roralpha). *J Biol Chem*. 2015; **290**(7): 4367-4382. doi: 10.1074/jbc.M114.605022
4. Leu DH, Draper BW. The ziwi Promoter Drives Germ line-Specific Gene Expression in Zebrafish. *Dev Dynam*. 2010; **239**(10): 2714-2721. doi: 10.1002/dvdy.22404
5. Leerberg DM, Sano K, Draper BW. Fibroblast growth factor signaling is required for early somatic gonad development in zebrafish. *PLoS Genet*. 2017; **13**(9): e1006993. doi: 10.1371/journal.pgen.1006993
6. Perz-Edwards A, Hardison NL, Linney E. Retinoic acid-mediated gene expression in transgenic reporter zebrafish. *Developmental biology*. 2001; **229**(1): 89-101. doi: 10.1006/dbio.2000.9979
7. Bunger MK, Wilsbacher LD, Moran SM *et al.* Mop3 is an essential component of the master circadian pacemaker in mammals. *Cell*. 2000; **103**(7): 1009-1017. doi: 10.1016/s0092-8674(00)00205-1
8. Storch KF, Paz C, Signorovitch J *et al.* Intrinsic circadian clock of the mammalian retina: importance for retinal processing of visual information. *Cell*. 2007; **130**(4): 730-741. doi: 10.1016/j.cell.2007.06.045
9. Mao X, Fujiwara Y, Chapdelaine A *et al.* Activation of EGFP expression by Cre-mediated excision in a new ROSA26 reporter mouse strain. *Blood*. 2001; **97**(1): 324-326. doi: 10.1182/blood.v97.1.324
10. Yoo SH, Yamazaki S, Lowrey PL *et al.* PERIOD2::LUCIFERASE real-time reporting of circadian dynamics reveals persistent circadian oscillations in mouse peripheral tissues. *Proc Natl Acad Sci U S A*. 2004; **101**(15): 5339-5346. doi: 10.1073/pnas.0308709101
11. Lee IT, Chang AS, Manandhar M *et al.* Neuromedin s-producing neurons act as essential pacemakers in the suprachiasmatic nucleus to couple clock neurons and dictate circadian rhythms. *Neuron*. 2015; **85**(5): 1086-1102. doi: 10.1016/j.neuron.2015.02.006
12. Xu Y, Toh KL, Jones CR *et al.* Modeling of a human circadian mutation yields insights into clock regulation by PER2. *Cell*. 2007; **128**(1): 59-70. doi: 10.1016/j.cell.2006.11.043
13. Sun Y, Zhang B, Luo L *et al.* Systematic genome editing of the genes on zebrafish Chromosome 1 by CRISPR/Cas9. *Genome Res*. 2019; **30**(1): 118-126. doi: 10.1101/gr.248559.119
14. Long Q, Meng A, Wang H *et al.* GATA-1 expression pattern can be recapitulated in living transgenic

zebrafish using GFP reporter gene. *Development*. 1997; **124**(20): 4105-4111. doi: 10.1242/dev.124.20.4105
15. Han B, Zhang Y, Bi X *et al.* Bi-FoRe: an efficient bidirectional knockin strategy to generate pairwise conditional alleles with fluorescent indicators. *Protein Cell*. 2021; **12**(1): 39-56. doi: 10.1007/s13238-020-00747-1
16. Hendel A, Bak RO, Clark JT *et al.* Chemically modified guide RNAs enhance CRISPR-Cas genome editing in human primary cells. *Nat Biotechnol*. 2015; **33**(9): 985-989. doi: 10.1038/nbt.3290
17. Yin L, Maddison LA, Li M *et al.* Multiplex Conditional Mutagenesis Using Transgenic Expression of Cas9 and sgRNAs. *Genetics*. 2015; **200**(2): 431-441. doi: 10.1534/genetics.115.176917
18. Leu DH, Draper BW. The ziwi promoter drives germline-specific gene expression in zebrafish. *Dev Dyn*. 2010; **239**(10): 2714-2721. doi: 10.1002/dvdy.22404
19. Brinkman EK, Chen T, Amendola M *et al.* Easy quantitative assessment of genome editing by sequence trace decomposition. *Nucleic Acids Res*. 2014; **42**(22): e168. doi: 10.1093/nar/gku936
20. Zhong Y, Ye Q, Chen C *et al.* Ezh2 promotes clock function and hematopoiesis independent of histone methyltransferase activity in zebrafish. *Nucleic acids research*. 2018; **46**(7): 3382-3399. doi: 10.1093/nar/gky101
21. Zhou L, Feng Y, Wang F *et al.* Generation of all-male-like sterile zebrafish by eliminating primordial germ cells at early development. *Scientific reports*. 2018; **8**(1): 1834. doi: 10.1038/s41598-018-20039-3
22. Welz PS, Zinna VM, Symeonidi A *et al.* BMAL1-Driven Tissue Clocks Respond Independently to Light to Maintain Homeostasis. *Cell*. 2019; **178**(4): 1029. doi: 10.1016/j.cell.2019.07.030
23. Liu K, Hou G, Wang X *et al.* Adverse effects of circadian desynchrony on the male reproductive system: an epidemiological and experimental study. *Human reproduction*. 2020; **35**(7): 1515-1528. doi: 10.1093/humrep/deaa101
24. Huang G, Zhang F, Ye Q *et al.* The circadian clock regulates autophagy directly through the nuclear hormone receptor Nr1d1/Rev-erbalpha and indirectly via Cebpb/(C/ebpbeta) in zebrafish. *Autophagy*. 2016; **12**(8): 1292-1309. doi: 10.1080/15548627.2016.1183843
25. Huang da W, Sherman BT, Lempicki RA. Systematic and integrative analysis of large gene lists using DAVID bioinformatics resources. *Nat Protoc*. 2009; **4**(1): 44-57. doi: 10.1038/nprot.2008.211
26. Zhou Y, Zhou B, Pache L *et al.* Metascape provides a biologist-oriented resource for the analysis of systems-level datasets. *Nat Commun*. 2019; **10**(1): 1523. doi: 10.1038/s41467-019-09234-6
27. Green CD, Ma Q, Manske GL *et al.* A Comprehensive Roadmap of Murine Spermatogenesis Defined by Single-Cell RNA-Seq. *Developmental cell*. 2018; **46**(5): 651-667 e610. doi: 10.1016/j.devcel.2018.07.025
28. Guo J, Grow EJ, Mlcochova H *et al.* The adult human testis transcriptional cell atlas. *Cell research*. 2018; **28**(12): 1141-1157. doi: 10.1038/s41422-018-0099-2
29. Wang Y, Wang R, Zhang S *et al.* iTALK: an R Package to Characterize and Illustrate Intercellular Communication. *bioRxiv*. 2019. doi: 10.1101/507871
30. Krzywinski M, Schein J, Birol I *et al.* Circos: an information aesthetic for comparative genomics. *Genome Res*. 2009; **19**(9): 1639-1645. doi: 10.1101/gr.092759.109
31. Rodriguez-Mari A, Canestro C, BreMiller RA *et al.* Retinoic acid metabolic genes, meiosis, and gonadal sex differentiation in zebrafish. *PLoS One*. 2013; **8**(9): e73951. doi: 10.1371/journal.pone.0073951
32. Huang J, Zhong Z, Wang M *et al.* Circadian modulation of dopamine levels and dopaminergic neuron development contributes to attention deficiency and hyperactive behavior. *The Journal of neuroscience : the official journal of the Society for Neuroscience*. 2015; **35**(6): 2572-2587. doi: 10.1523/JNEUROSCI.2551-14.2015

33. Liu C, Hu J, Qu C *et al.* Molecular evolution and functional divergence of zebrafish (Danio rerio) cryptochrome genes. *Scientific reports*. 2015; **5**: 8113. doi: 10.1038/srep08113
34. Nakagata N. Cryopreservation of mouse spermatozoa and in vitro fertilization. *Methods in molecular biology*. 2011; **693**: 57-73. doi: 10.1007/978-1-60761-974-1_4
35. Wu G, Anafi RC, Hughes ME *et al.* MetaCycle: an integrated R package to evaluate periodicity in large scale data. *Bioinformatics*. 2016; **32**(21): 3351-3353. doi: 10.1093/bioinformatics/btw405
36. Zielinski T, Moore AM, Troup E *et al.* Strengths and limitations of period estimation methods for circadian data. *PLoS One*. 2014; **9**(5): e96462. doi: 10.1371/journal.pone.0096462
37. Zhang R, Podtelezhnikov AA, Hogenesch JB *et al.* Discovering Biology in Periodic Data through Phase Set Enrichment Analysis (PSEA). *J Biol Rhythms*. 2016; **31**(3): 244-257. doi: 10.1177/0748730416631895
38. Singer JM, Hughey JJ. LimoRhyde: A Flexible Approach for Differential Analysis of Rhythmic Transcriptome Data. *J Biol Rhythms*. 2019; **34**(1): 5-18. doi: 10.1177/0748730418813785
